# Rare Functional Variant in *TM2D3* is Associated with Late-Onset Alzheimer's Disease

**DOI:** 10.1371/journal.pgen.1006327

**Published:** 2016-10-20

**Authors:** Johanna Jakobsdottir, Sven J. van der Lee, Joshua C. Bis, Vincent Chouraki, David Li-Kroeger, Shinya Yamamoto, Megan L. Grove, Adam Naj, Maria Vronskaya, Jose L. Salazar, Anita L. DeStefano, Jennifer A. Brody, Albert V. Smith, Najaf Amin, Rebecca Sims, Carla A. Ibrahim-Verbaas, Seung-Hoan Choi, Claudia L. Satizabal, Oscar L. Lopez, Alexa Beiser, M. Arfan Ikram, Melissa E. Garcia, Caroline Hayward, Tibor V. Varga, Samuli Ripatti, Paul W. Franks, Göran Hallmans, Olov Rolandsson, Jan-Håkon Jansson, David J. Porteous, Veikko Salomaa, Gudny Eiriksdottir, Kenneth M. Rice, Hugo J. Bellen, Daniel Levy, Andre G. Uitterlinden, Valur Emilsson, Jerome I. Rotter, Thor Aspelund, Cohorts for Heart and Aging Research in Genomic Epidemiology consortium, Alzheimer’s Disease Genetic Consortium, Genetic and Environmental Risk in Alzheimer’s Disease consortium, Christopher J. O’Donnell, Annette L. Fitzpatrick, Lenore J. Launer, Albert Hofman, Li-San Wang, Julie Williams, Gerard D. Schellenberg, Eric Boerwinkle, Bruce M. Psaty, Sudha Seshadri, Joshua M. Shulman, Vilmundur Gudnason, Cornelia M. van Duijn

**Affiliations:** 1 Icelandic Heart Association, Kopavogur, Iceland; 2 Department of Epidemiology, Erasmus University Medical Center, Rotterdam, The Netherlands; 3 Cardiovascular Health Research Unit, Department of Medicine, University of Washington, Seattle, Washington, United States of America; 4 Boston University School of Medicine, Boston, Massachusetts, United States of America; 5 Framingham Heart Study, Framingham, Massachusetts, United States of America; 6 Department of Molecular and Human Genetics, Baylor College of Medicine, Houston, Texas, United States of America; 7 Jan and Dan Duncan Neurological Research Institute, Texas Children’s Hospital Houston, Texas, United States of America; 8 Program in Developmental Biology, Baylor College of Medicine, Houston, Texas, United States of America; 9 School of Public Health, Human Genetics Center, The University of Texas Health Science Center at Houston, Houston, Texas, United States of America; 10 Department of Biostatistics and Epidemiology, Perelman School of Medicine, University of Pennsylvania, Philadelphia, Pennsylvania, United States of America; 11 Institute of Psychological Medicine and Clinical Neurosciences, Medical Research Council (MRC) Centre for Neuropsychiatric Genetics & Genomics, Cardiff University, Cardiff, United Kingdom; 12 Department of Biostatistics, Boston University School of Public Health, Boston, Massachusetts, United States of America; 13 Faculty of Medicine, University of Iceland, Reykjavik, Iceland, United States of America; 14 Department of Neurology, Erasmus University Medical Center, CA Rotterdam, The Netherlands; 15 Department of Neurology, University of Pittsburgh Medical Center, Pittsburgh, Pennsylvania, United States of America; 16 Departments of Radiology, Erasmus University Medical Center, CA Rotterdam, The Netherlands; 17 Laboratory of Epidemiology and Population Sciences, National Institute on Aging, Bethesda, Maryland, United States of America; 18 MRC Human Genetics Unit, Institute of Genetics and Molecular Medicine, University of Edinburgh, Edinburgh, United Kingdom; 19 Generation Scotland, Centre for Genomic and Experimental Medicine, University of Edinburgh, Edinburgh, United Kingdom; 20 Department of Clinical Sciences, Genetic and Molecular Epidemiology Unit, Lund University, Malmö, Sweden; 21 Institute for Molecular Medicine Finland (FIMM), University of Helsinki, Helsinki, Finland; 22 Department of Public Health, University of Helsinki, Helsinki, Finland; 23 Department of Public Health & Clinical Medicine, Umeå University Hospital, Umeå, Sweden; 24 Department of Nutrition, Harvard T.H. Chan School of Public Health, Boston, Massachusetts, United States of America; 25 Department of Biobank Research, Umeå University, Umeå, Sweden; 26 Department of Public Health & Clinical Medicine, Section for Family Medicine, Umeå University, Umeå, Sweden; 27 Research Unit, Skellefteå Hospital, Skellefteå, Sweden; 28 Centre for Genomic and Experimental Medicine, Institute of Genetics and Molecular Medicine, University of Edinburgh, Edinburgh, United Kingdom; 29 National Institute for Health and Welfare, Helsinki, Finland; 30 Department of Biostatistics, University of Washington, Seattle, Washington, United States of America; 31 Howard Hughes Medical Institute, Durham, North Carolina, United States of America; 32 Division of Intramural Research, National Heart, Lung, and Blood Institute, National Institutes of Health, Bethesda, Maryland, United states of America; 33 Department of Internal Medicine, Erasmus University Medical Center, CA Rotterdam, The Netherlands; 34 Faculty of Pharmaceutical Sciences, University of Iceland, Reykjavik, Iceland; 35 Institute for Translational Genomics and Population Sciences, Los Angeles BioMedical Research Institute and Departments of Medicine and Pediatrics, Harbor-UCLA Medical Center, Torrance, California, United States of America; 36 Centre for Public Health, University of Iceland, Reykjavik, Iceland; 37 Department of Epidemiology, University of Washington, Seattle, Washington, United States of America; 38 Collaborative Health Studies Coordinating Center, Seattle, Washington, United States of America; 39 Department of Pathology and Laboratory of Medicine, Perelman School of Medicine, University of Pennsylvania, Philadelphia, Pennsylvania, United States of America; 40 Human Genome Sequencing Center, Baylor College of Medicine, Houston, Texas, United States of America; 41 Department of Health Services, University of Washington, Seattle, Washington, United States of America; 42 Group Health Research Institute, Group Health Cooperative, Seattle, Washington, United States of America; Case Western Reserve University, UNITED STATES

## Abstract

We performed an exome-wide association analysis in 1393 late-onset Alzheimer’s disease (LOAD) cases and 8141 controls from the CHARGE consortium. We found that a rare variant (P155L) in *TM2D3* was enriched in Icelanders (~0.5% versus <0.05% in other European populations). In 433 LOAD cases and 3903 controls from the Icelandic AGES sub-study, P155L was associated with increased risk and earlier onset of LOAD [odds ratio (95% CI) = 7.5 (3.5–15.9), p = 6.6x10^-9^]. Mutation in the *Drosophila TM2D3* homolog, *almondex*, causes a phenotype similar to loss of Notch/Presenilin signaling. Human *TM2D3* is capable of rescuing these phenotypes, but this activity is abolished by P155L, establishing it as a functionally damaging allele. Our results establish a rare *TM2D3* variant in association with LOAD susceptibility, and together with prior work suggests possible links to the β-amyloid cascade.

## Author Summary

Alzheimer’s disease (AD) is the most common cause of dementia in the older adult population. There is substantial evidence for an important genetic contribution to AD risk. While prior work has comprehensively evaluated the contribution of common genetic variants in large population-based cohorts, the role of rare variants remains to be defined. Here, we have used a newer genotyping array to characterize less common variants, including those likely to impact the function of encoded proteins, in a combined cohort of 1393 AD cases and 8141 control subjects without AD. Our results implicate a novel, amino acid-changing variant, P155L, in the *TM2D3* gene. This variant was discovered to be more common in the Icelandic population, where it was significantly associated with both increased risk and earlier age of onset of AD. Lastly, in order to examine the potential functional impact of the implicated variant, we performed additional studies in the fruit fly. Our results suggest that P155L causes a loss-of-function in TM2D3, in the context of Notch-Presenilin signal transduction. In sum, we identify a novel, rare *TM2D3* variant in association with AD risk and highlight functional connections with AD-relevant biology.

## Introduction

Alzheimer’s disease (AD, [MIM: 104300]), the most common form of dementia, affects more than 10% of those 65 years and older, increasing to 30% in those 85 years and older [1–4]. Pathologically, AD is characterized by extensive brain neurodegenerative cell loss in association with extra-cellular β-amyloid plaques and intra-neuronal tangles consisting of hyper-phosphorylated tau protein. Multiple mutations in the amyloid-β precursor protein (*APP*, [MIM: 104760]) and the presenilin-1 and -2 (*PSEN1* [MIM: 104311] and *PSEN2* [MIM: 600759]) genes cause familial early-onset (<65 years) AD [5,6]. Genome-wide association studies (GWAS) have also identified numerous common genetic variants of modest effect sizes for late-onset AD (LOAD, [MIM: 104300]) [7–12]. However, *Apolipoprotein E* (*APOE*, [MIM: 107741]*)* still remains the most important known genetic determinant of LOAD susceptibility [13,14]. Recently, rare variants with effect sizes similar to the *APOE*-ε4 allele have also been identified. While the population-attributable risk of such single rare variants is low, their discoveries may have important implications for understanding disease mechanisms and developing novel treatments [15]. Recent examples include a rare protective allele of *APP* [16] as well as variants in the novel genes *TREM2* [MIM: 605086] [17,18], *PLD3* [MIM: 615698] [19], *UNC5C* [MIM: 603610] [20], and *AKAP9* [MIM:604001] [21]. These findings highlight potentially important, new cellular pathways relevant for disease pathophysiology [15]; for example *TREM2* has spurred intense recent interest in the role of microglia in LOAD [22,23]. Importantly, because of population histories and demography, rare variants may be population-specific. The protective *APP* variant, for example, is found predominantly in Iceland and other Scandinavian populations [16]. The two variants in *AKAP9* have only been reported in African-Americans [21], and *PLD3* variants appear to vary greatly in frequency between populations [24].

To date, the identification of rare susceptibility variants in LOAD has been hampered by poor representation on genotyping arrays used for large GWAS [25]; moreover, direct sequencing in large numbers of individuals remains costly. Here, using an exome-wide genotyping array (the Illumina HumanExome Beadchip), we report associations with LOAD in four population-based cohorts from the Cohorts for Heart and Aging Research in Genomic Epidemiology (CHARGE) consortium [26].

## Results

### Exome-wide association study of LOAD in CHARGE

The discovery phase of our analysis examined single-variant associations with LOAD. Meta-analysis was performed across four CHARGE studies including 1393 LOAD cases and 8141 controls (**Table 1**), using logistic regression-based scores [27,28] and adjusting for age and sex (see **Methods** and **Supporting methods in S1 Text**). Single-variant results were filtered using quality control filters that included minimum minor allele frequency (MAF ≥ 0.5%) or minor allele count (MAC ≥ 5) in cases (see also **Methods**) resulting in 52026 total variants tested. Given the rarity of many variants captured on the exome array, we additionally performed association analysis, where we considered rare variants from the same gene in aggregate. For this complementary analysis we used the Sequence Kernel Association Test (SKAT) test [29], a variance component score test for the association of a set of multiple variants with a trait. SKAT tests the null hypothesis of no variation of effects and therefore the statistical model of SKAT is applicable to test association of variant sets that may have a combination of variants including both risk and protective alleles or those with no effect. Variants were included in the SKAT analysis based on functional annotation (see **Methods**) and maximum frequency of 5%. Because the SKAT results were filtered by cumulative set-based MAF and MAC, considering all variants in aggregate, and not by minimum MAF of single SNPs (see **Methods**), this complementary analysis included many variants that did not meet the criteria for inclusion in the single-variant tests. The results of both the single-variant and SKAT discovery analysis are presented in **S1 Table** and quantile-quantile plots are in **S1 Fig**. Below, we discuss each of the top three results, our efforts to replicate the novel loci in independent cohorts, and for *TM2D3*, subsequent functional validation of the implicated variant in *Drosophila*.

**Table 1:** Sample characteristics of discovery cohorts.

	AGES		FHS		CHS		RS	
Characteristics	Controls	Cases	Controls	Cases	Controls	Cases	Controls	Cases
Study Design	Population-based cohort		Population-based cohort		Population-based cohort		Population-based cohort	
No. participants	2374	143	1338	230	2013	557	2416	463
Women, No (%)	1401 (59)	85 (59)	754 (56)	158 (69)	1134 (56)	343 (62)	1227(51)	319(69)
Age, mean (SD), year	78.89 (4.98)	82.50 (4.94)	79.84 (8.57)	85.06 (6.90)	81.18 (5.15)	82.1 (5.32)	78.2(7.71)	83.3(6.59)
*APOE* ε4+, No. (%)	638 (27)	66 (46)	258 (20)	74 (32)	397 (20)	176 (32)	609(26)	190(43)

AGES: Age, Gene/Environment Susceptibility study, FHS: Framingham Heart Study, CHS: Cardiovascular Health Study, RS: Rotterdam Study.

### APOE

As expected based on many prior LOAD GWAS [12], three common intronic variants near *APOE* reached the Bonferroni threshold of exome-wide significance (p< = 9.6x10^-7^) in the single-variant analysis: rs769449 in *APOE* (p = 5.8x10^-38^, MAF_case_ = 17%, MAF_control_ = 10%), rs2075650 in *TOMM40* (p = 1.2x10^-24^, MAF_case_ = 18%, MAF_control_ = 13%), and rs6859 in *PVRL2* (p = 1.5x10^-9^, MAF_case_ = 46%, MAF_control_ = 40%). Although *APOE* is a well-established LOAD risk locus it did not achieve nominal significance in the SKAT analysis, possible because the established common risk variants at this locus exceed our frequency threshold for consideration in the SKAT analysis (MAF<0.05) (**S2 Table**).

### SKAP2

rs17154402 in *SKAP2* (MIM: 605215) on chromosome 7p15 also showed a significant association with LOAD (p = 2.1x10^-7^, MAF_case_ = 0.22%, MAF_control_ = 0%). This SNP is predicted to introduce a non-synonymous amino acid change (S253T in the longer isoform) in the SKAP2 protein. The *SKAP2* locus also achieved significant association with LOAD (p = 4.5x10^-7^) in the SKAT analysis. Although the SKAT analysis included three distinct *SKAP2* missense alleles, the SKAT result was fully explained by the variant identified in the single-variant analysis (**S2 Table**). rs17154402 was not significantly associated with LOAD risk in the ADGC or GERAD cohorts, which are 2 large European-ancestry case-control datasets (13 carriers in 8256 controls [MAF 0.08%] and 9 carriers in 13333 cases [MAF 0.03%], p = 0.05), and compared to the discovery CHARGE cohort, showed an opposite direction of effect in these samples. We noted that rs17154402 has a higher frequency in persons of African compared to European ancestry (MAF~7% in Yorubans and 3% in all African populations combined in the 1000 Genomes Project data, and Grove et al [30] reported a ~5% MAF in African populations versus 0.22% in European ancestry subjects). We therefore took advantage of an available African-American subsample of the CHARGE CHS cohort for attempted replication. However, the variant again showed an inconsistent direction of effect and non-significant association (93 [12 carriers] cases, 208 [33 carriers] controls; p = 0.66). Based on the non-replication of *SKAP2* in available independent datasets, it was not pursued further.

### TM2D3

Our discovery analysis also identified rs139709573, a missense variant in *TM2D3* (MIM: 610014) on chromosome 15q26. Although the association statistic did not exceed the Bonferroni threshold for exome-wide significance in the CHARGE discovery analysis (single-variant p = 2.0x10^-6^, SKAT p = 8.3x10^-6^), several important observations led us to consider this variant further. First, we noted that while very rare (0–0.06% MAF in European ancestry populations (**S3 Table**)) the *TM2D3* variant was enriched nearly 10-fold in the Icelandic AGES cohort (0.45% MAF). Second, rs139709573 was significantly associated (p = 5.9x10^-8^, **Table 2**) with LOAD when the analysis was restricted to the AGES-discovery sample (143 cases, 2374 controls). Third, prior experimental studies potentially link *TM2D3* to AD-relevant biology. Specifically, *TM2D3* shares homology with the β-amyloid peptide binding protein (BBP or *TM2D1*) [31], and as discussed further below, genetic studies of the conserved *Drosophila* ortholog almondex (*amx*) strongly suggest links to γ-secretase function [32].

**Table 2:** Sample characteristics and association results for P155L (rs139709573) in *TM2D3* in the two Icelandic AGES cohorts.

Cohort	Group	No(% women)	Age, mean (SD), year	*APOE* ε4+ No (%)	*TM2D3* carriers, No (%)	p (OR, 95% CI)^a^	p_Fisher’s Exact_^b^	p_conditional_^c^
AGES-discovery	Cases	143 (59)	82.5 (4.9)	66 (46)	7 (4.9)	5.9x10^-8^ (8.62, 3.43–21.68)	5.6x10^-4^	8.4x10^-8^
	Controls	2374 (59)	78.9 (5.0)	638 (27)	20 (0.8)	…	…	…
AGES-followup	Cases	290 (59)	84.5 (5.1)	127 (44)	6 (2.1)	3.0x10^-3^ (5.42, 1.60–18.32)	6.2x10^-3^	1.1x10^-2^
	Controls	1529 (57)	79.4 (5.3)	396 (26)	6 (0.4)	…	…	…
Pooled^d^	Cases	433 (59)	83.9 (5.2)	193 (45)	13 (3.0)	6.6x10^-9^ (7.45, 3.49–15.90)	5.9x10^-5^	6.8x10^-8^
	Controls	3903 (58)	79.1 (5.1)	1034 (26)	26 (0.7)	…	…	…

All p-values are based on the 2-sided alternative.

a Score tests adjusted for age and sex based on a logistic regression model. The unconditional MLE of the OR is reported based on fitting the full model including the SNP. Note, that those point estimates of OR could be inflated (**Supporting results in S1 Text**)

b P-value from the Fisher's exact test for carrier status. For the meta-analysis the data were pooled.

c P-value conditional on *APOE* ε4 carrier status. Based on a score test after adjusting for age, sex, and *APOE* ε4.

d The two cohorts are pooled in a stratified analysis (stratified by cohort).

**Fig 1:**
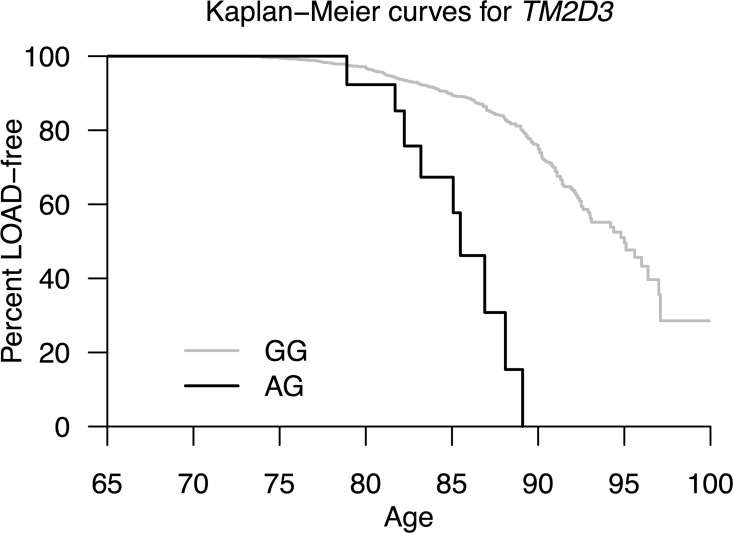
Kaplan-Meier survival curves for *TM2D3*. Curves are based on incident data only. There was no evidence of bias due to competing risk of death (**Supporting results in S1 Text**).

In the SKAT analysis, the association of *TM2D3* with LOAD was fully accounted for by rs139709573; although, 3 other variants were considered in this analysis (**S2 Table**). In the subsequent analyses and functional follow-up, we therefore restricted our focus on rs139709573. First we evaluated potential inflation in the test statistic of the *TM2D3* rs139709573 result in the AGES-discovery cohort (**Supporting results in S1 Text**) and we further confirmed the genotypes of risk allele carriers by re-genotyping them with a TaqMan assay. We next directly genotyped rs139709573 in an independent Icelandic AGES-followup cohort (290 cases, 1529 controls), consisting of individuals who had not been genotyped previously with the exome array. The rs139709573 association was also detected in this sample (p = 3.1x10^-3^, **Table 2**). As expected, an analysis combining all of the AGES data on *TM2D3* demonstrated an enhanced association of the *TM2D3* variant with LOAD (OR = 7.45; 95% CI 3.49–15.90; p = 6.6x10^-9^). We also observed an association with age-at-onset in the AGES cohorts (hazard ratio = 5.3; 95% CI 2.7–10.5; p = 1.1x10^-6^, see also non-parametric Kaplan-Meier curves in **Fig 1**). Using the PHASE program [33–35] we estimated that rs139709573 resides on a single haplotype in the Icelandic population (**Supporting results in S1 Text**), and we further excluded potential confounding due to cryptic relatedness (**Supporting results in S1 Text**). Similarly, the association between rs139709573 and LOAD was robust to adjustment for *APOE* genotype (**Table 2**). Given the low frequency of rs139709573 in individuals of European ancestry, there were limited numbers of carriers in GERAD and ADGC (11 carriers in 13333 cases [0.041% MAF]; 4 carriers in 8256 controls [0.024% MAF]). While, the association was not statistically significant (p = 0.4), the direction of effect was consistent with that observed in AGES (OR = 1.7, 95% CI 0.5–7.3).

### Analysis of *TM2D3* transcripts and expression

The *TM2D3* gene has several alternatively spliced transcripts, including six protein-coding transcripts [36]. rs139709573 falls within an exon common to all six isoforms (**S2 Fig**) and causes a Proline to Leucine amino acid change (P155L in the longest isoform). Gene and transcript expression estimates were extracted from publically available Genotype-Tissue Expression consortium (GTEx) data [37], estimated as “transcripts per million” (TPM), using the Toil workflow [38] in the UCSC Xena browser (http://xena.ucsc.edu/). *TM2D3* is expressed in all GTEx tissues, including from many brain regions (**S3 Fig**). The GTEx expression data further show that the alternative *TM2D3* transcripts are similarly expressed across diverse human tissues (**S4 Fig**).

### Structure of TM2D3 protein

**Fig 2:**
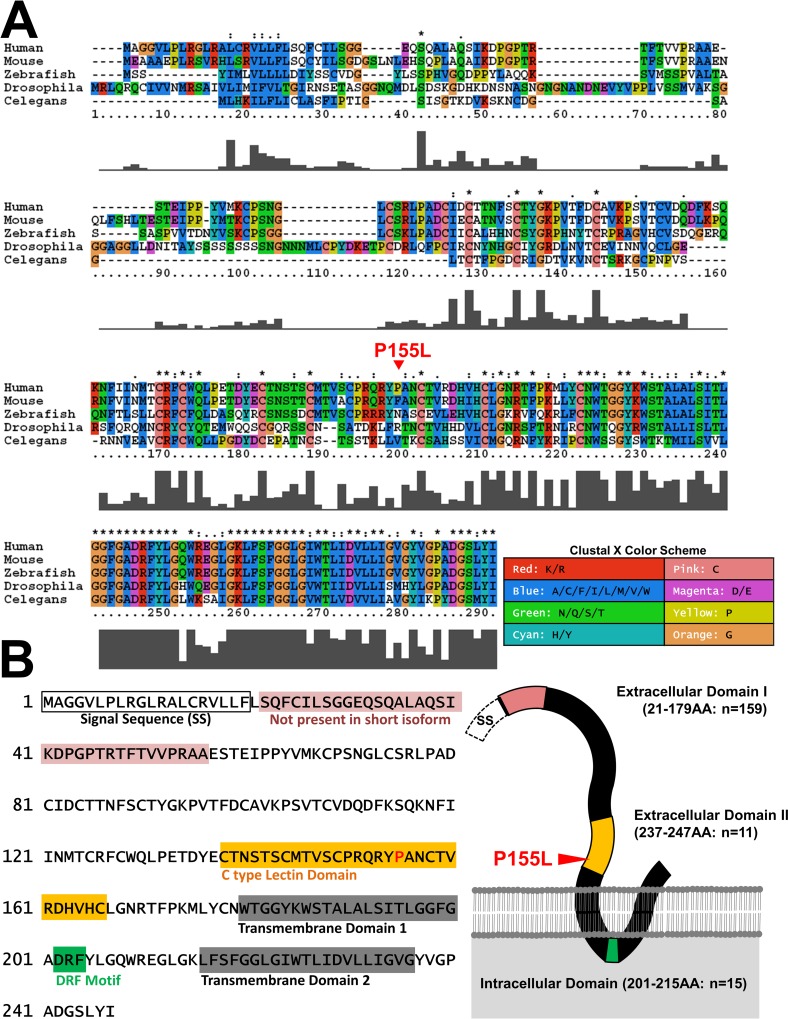
Sequence alignment and domain structure of TM2D3. (A) Sequence alignment of TM2D3 homologs shows overall strong conservation but lack of conservation for P155L. Sequence alignment of human (TM2D3), mouse (Tm2d3), zebrafish (tm2d3), *Drosophila* (amx) and *C*. *elegans* (C41D11.9) using Clustal X2.1 is shown. The conserved amino acids are highlighted according to the standard color scheme of Clustal X. (B) Primary sequence and schematic diagram of the domain structure of human TM2D3.

*TM2D3* encodes a predicted double-pass transmembrane protein with evidence of evolutionary conservation (**Fig 2A**). The variant falls within the overall well-conserved C-Type Lectin domain and is adjacent to other invariant residues in the first predicted extracellular domain [32] (**Fig 2B**). While P155L is not predicted to be strongly damaging by PolyPhen [39], SIFT [40], or CADD (C-Score = 7.3) [41] and cross-species alignments show that the Proline residue is not conserved (**Fig 2A**), the two amino-acids do have important property differences, consistent with a potentially non-conservative substitution. Proline, the only cyclic amino-acid, frequently resides on the surface of folded proteins, and can underlie structural “kinks” or bends. Proline is unique in its inability to form hydrogen bonds that stabilize alpha-helices and beta-sheets. By contrast with Proline, Leucine favors alpha-helical secondary structure, and is more commonly buried in the interior of folded protein structures [42].

### Functional validation of *TM2D3* (P155L) in *Drosophila*

**Fig 3:**
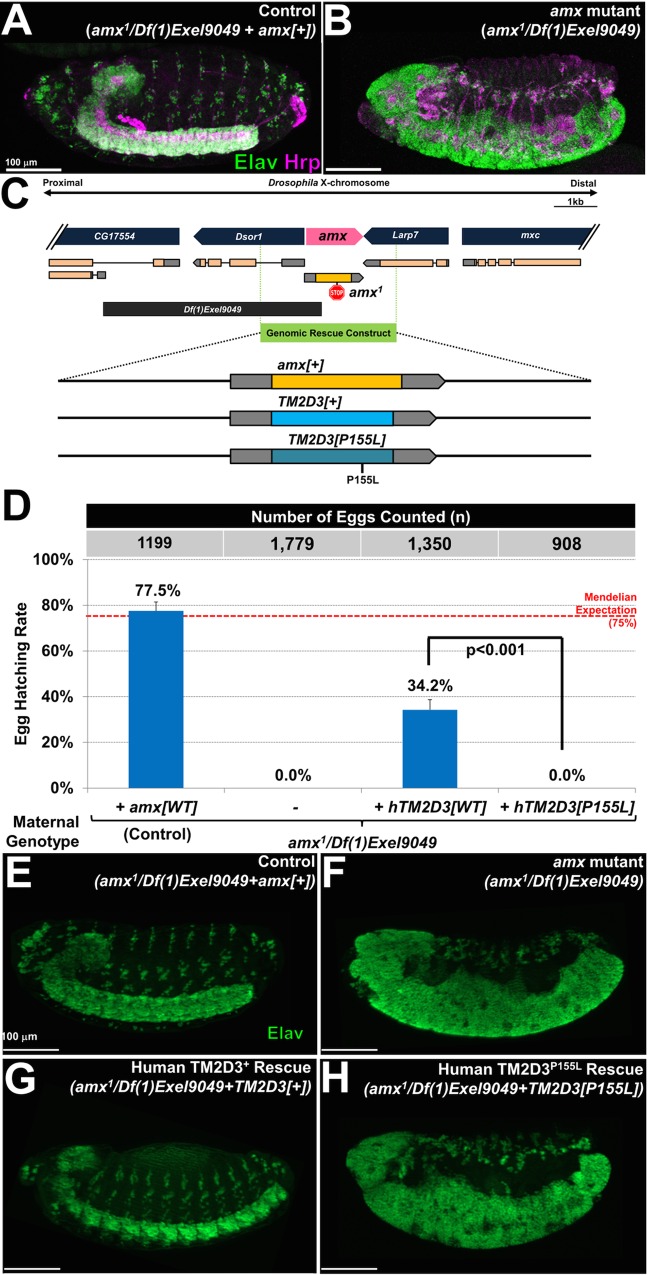
*TM2D3*^*P155L*^ is a loss-of-function allele. (A-B) Embryos laid by *amx* mutant females exhibit a strong neurogenic phenotype. (A) Lateral view of a late stage control embryo shows properly patterned and organized central and peripheral nervous system structures. (B) Embryos laid by amx mutant females show dramatic increase in the number of neurons, labeled by Elav (neuronal nucleus, green) and Hrp (neuronal membrane, magenta). (C) Schematic diagram of the *amx* locus and genomic rescue constructs generated for this study. *amx*^*1*^ contains an 8 nucleotide deletion that introduces a frameshift followed by a stop codon after residue 184 (Michellod and Randsholt, 2008). *Df(1)Exel9049* is a molecularly defined deletion that covers *amx* and two neighboring genes. The genomic rescue construct contains a ~3.3 kb fragment that can fully rescue the sterility of *amx*^*1*^*/Df(1)Exel9049* mutant females and the neurogenic defects seen in the progeny (A, E). Coding region of Amx has been replaced by TM2D3 to “humanize” the fly *amx* gene. (D) Egg hatching assay reveals that *hTM2D3[+]* can partially suppress the female sterility of *amx*^*1*^*/Df(1)Exel9049* mutant females, while *hTM2D3[P155L]* cannot. Due to the lethality of *Df(1)Exel9049/Y* hemizygous male progeny, complete rescue of the *amx* phenotype is expected to lead to a maximum of ~75% egg hatching, as denoted by the Mendelian Expectation line (also see S3 Fig). (E-H) The developing nervous system is shown for embryos laid by *amx* mutant females with and without *amx[+]*, *TM2D3[+]*, and *TM2D3[P155L]* genomic rescue constructs. *TM2D3[+]* is capable of complete rescue of the *amx* neurogenic phenotype in some embryos (G), whereas all embryos with the *hTM2D3[P155L]* construct exhibit strong neurogenic phenotypes (H). Also see S5 Fig for assessment of rescue of the *amx* peripheral nervous system neurogenic phenotype. Scale bars = 100μm.

In order to investigate the potential functional consequences of P155L and its possible link to AD, we turned to the fruit fly, *Drosophila melanogaster*. Human *TM2D3* and the homologous fly gene, *amx*, are 51% identical and 64% similar (**Fig 2A**). Mutations in *amx* cause a strong maternal effect neurogenic phenotype, characteristic of defective Notch signaling, and lead to embryonic lethality (**Fig 3A and 3B**) [43,44]. Similar to the cleavage of APP to generate the pathogenic ß-amyloid peptide, Notch receptor signaling requires homologous cleavage by γ-secretase for receptor activation. In *Drosophila*, loss of *Notch* or its regulators (e.g. *presenilin*) cause similar neurogenic phenotypes, due to impaired lateral inhibition and the inappropriate differentiation of ectodermal tissue toward a neural fate [45–47]. Moreover, prior genetic epistasis experiments suggest that *amx* may function at the γ-secretase cleavage step [32].

To determine whether *TM2D3* and *amx* have conserved molecular functions, we “humanized” the *Drosophila amx* gene by replacing its coding sequence with that of human *TM2D3* in the context of a genomic rescue construct (**Fig 3C**). Following establishment of stable transgenic lines, this construct was crossed into an *amx* mutant genetic background (*amx*^*1*^*/Df(1)Exel9049)*. Consistent with prior reports [43,44], when crossed to *amx* mutant males, all embryos laid by *amx* mutant females exhibited a strong neurogenic phenotype, failing to hatch from their eggs (**Fig 3B and 3D**). This embryonic defect and the resulting female sterility can be fully rescued by a genomic rescue construct with the wild-type (*+*) fly *amx* gene (**Fig 3D and 3E**). Due to the lethality of *Df(1)Exel9049/Y* hemizygous male progeny, complete rescue of the *amx* phenotype is predicted to lead to a maximum of ~75% egg hatching based on Mendelian expectations (**Fig 3D, S5 Fig**). Introduction of wild-type human *TM2D3*, under control of endogenous *amx* regulatory sequences, demonstrated significant rescue activity compared to *amx* mutant females (34.2% vs. 0% egg hatching p<0.001). Overall, based on the egg hatching assay (**Fig 3D**), the activity of the human *TM2D3* construct was estimated to be roughly half that observed for fly *amx*. Consistent with this, we observed a range in the severity of the neurogenic phenotype severity (**S6 Fig**), including embryos exhibiting a complete rescue (**Fig 3G**) and producing viable progeny that can develop into adult flies with no obvious morphological phenotypes. By contrast, an otherwise identical *TM2D3*^*P155L*^ genomic construct, but harboring the AD-associated P155L variant, was unable to rescue the neurogenic phenotype (**Fig 3H**) or the associated female sterility (**Fig 3D**). No animals hatched out of more than 900 eggs laid from *TM2D3*^*P155L*^ female flies. Consistent results were also seen in complementary assessments of rescue activity for the *amx* peripheral nervous system neurogenic phenotype (**S6 Fig**). In sum, our results demonstrate that human *TM2D3* can functionally substitute for fly *amx* in the context of embryonic Notch signaling, and that the P155L variant causes a loss-of-function in this context.

## Discussion

We identified a novel LOAD-associated gene, *TM2D3*, harboring a rare missense mutation (P155L) that is associated with increased risk and earlier onset of LOAD diagnosis in an Icelandic population. Our experiments in *Drosophila* further suggest that this LOAD-associated P155L variant leads to a loss-of-function of TM2D3 in the context of Notch signaling during embryogenesis. While we could neither confirm nor negate the *TM2D3* finding in available European ancestry populations–possibly because of the low P155L allele frequency, a consistent association was demonstrated in an Icelandic cohort. Although the *TM2D3* variant failed to achieve the Bonferroni significance threshold in our CHARGE-wide meta-analysis our observation of increased frequency and enhanced association within the Icelandic AGES-discovery cohort highlighted the TM2D3 variant for further consideration. Significant association in the AGES-followup sample coupled with variant functional validation in *Drosophila* suggests a role for TM2D3 in AD susceptibility. Our investigation thus exemplifies the importance not only for statistical rigor and transparency, but also flexibility in study design, when performing rare variant genetic association studies, in particular when statistical results are accompanied by relevant functional experiments.

*TM2D3* has not previously been directly linked to AD. The encoded protein is predicted to contain two transmembrane regions, and additional domains with homology to C-Type lectins and G-protein coupled receptors (DRF motif) (**Fig 2B**). Since the P155L variant resides within the well-conserved C-Type lectin domain, the variant could plausibly disrupt the interaction of TM2D3 with a putative glycosylated binding partner. Alternatively, as suggested above, distinct structural properties of Leucine versus Proline may disrupt protein folding [42]. Interestingly, *TM2D3* shares homology with the β-amyloid peptide binding protein (BBP or *TM2D1*), which avidly binds to and sensitizes cells to toxicity caused by the β-amyloid peptide [31]. However, overexpression of TM2D3 (referred to as BLP2 in Kajkowski et al. [31]) was not associated with similar toxicity in cell culture. In mammals, TM2D3 it is highly expressed in the brain, including in the hippocampus, hypothalamus, and amygdala, potentially consistent with a role in AD pathogenesis (http://biogps.org/#goto=genereport&id=80213 and **S3 Fig**) [31,48,49]. Additionally, micro-array analysis suggests that the expression of *TM2D3* is down-regulated in the hippocampus of AD cases compared to controls [50].

While incompletely studied in mammals, loss-of-function mutants in the well-conserved *Drosophila* homolog of *TM2D3*, *amx*, cause embryonic phenotypes associated with disruptions in Notch signaling [51]. Further, genetic epistasis experiments support a potential role for *amx* in Notch receptor intramembranous proteolysis [32], a process that is mediated by the presenilin/γ-secretase complex. One speculative hypothesis is that TM2D3/Amx participates either directly or indirectly in the intramembranous cleavage of Notch as well as in the homologous proteolytic processing of APP by γ-secretase. In this model, the P155L variant might potentially influence the generation of toxic β-amyloid peptides, similar to well-established disease susceptibility variants in *APP*, *PSEN1*, and *PSEN2* [52].

Taking advantage of the potential functional conservation, we found that human TM2D3 expressed under the control of endogenous *Drosophila amx* regulatory elements is capable of rescuing the embryonic neurogenic phenotype, indicating that Amx and TM2D3 are orthologs and have conserved molecular functions *in vivo*. Importantly, we found that introduction of the LOAD-associated P155L variant into an otherwise identical *TM2D3* genomic rescue construct abolishes the rescue activity. This result is consistent with either a partial or complete loss-of-function mechanism of the P155L variant, albeit within the heterologous context of Notch signaling in the *Drosophila* embryo. It is notable that the P155L variant was neither well-conserved nor predicted by bioinformatics as damaging; nevertheless, our cross-species approach convincingly demonstrates its functional impact. Our overall strategy of first rescuing the loss-of-function phenotype of the predicted homologous gene in flies, followed by examining the potential consequences of an implicated variant can be a powerful approach for the follow-up of many similar findings emerging from human genomic studies, including others using exome genotyping arrays or next generation sequencing approaches [53,54].

In summary, we have identified a missense mutation in the *TM2D3* gene with a strong impact on LOAD risk. The *TM2D3* variant is enriched ~10-fold and associated with both risk and age-at-onset of LOAD in the Icelandic population. We further show that P155L is associated with a loss-of-function in the heterologous but potentially relevant context of Notch signaling in *Drosophila* embryos. We therefore speculate that TM2D3 may participate in the proteolytic processing of both Notch and APP, linking it to the amyloid cascade like other well-established AD susceptibility variants. Although we have demonstrated an association of the *TM2D3* variant only in the Icelandic population, our findings may thus have broader implications for understanding LOAD.

## Methods

### Studies and participants

Four studies from the CHARGE consortium genotyped a total of 1393 AD cases and 8141 cognitively intact controls for an ExomeChip (EC) genotyping array. All participants in the discovery phase of the analysis were of European or European American descent (**Table 1**). In the follow-up analysis we included Icelandic individuals from the AGES-followup cohort, African-American participants of the Cardiovascular Health Study (CHS), and European or European American individuals from the Alzheimer’s Disease Genetics Consortium (ADGC) and Genetic and Environmental Risk in Alzheimer’s Disease consortium (GERAD). Further details on phenotyping and other cohort characteristics are in the **Supporting methods in S1 Text**.

### Ethics statement

All participants provided informed consent and all studies were approved by their respective ethics committees.

### Genotyping

All four CHARGE cohorts were genotyped for the HumanExome BeadChip v1.0 from Illumina (San Diego, CA, USA). Genotype calling and quality control was performed centrally as described previously [30]. Each study also performed quality control locally (**Supporting methods in S1 Text**). Post-analysis quality control on the top results included visual inspection of cluster plots (**S1 Fig**) and re-genotyping of *TM2D3* carriers using a TaqMan assay (genotype calls were 100% validated). *TM2D3* variant was genotyped in the AGES-followup cohort using the TaqMan assay. In ADGC and GERAD consortia the *SKAP2* and *TM2D3* variants were gentoyped on the Illumina HumanExome BeadChip v1.0 or v1.1 from Illumina (**Supporting methods in S1 Text**).

### Statistical analysis

#### Statistical methods of the discovery phase

In the discovery phase of our analysis we used score tests [27] for the single-variant analysis and the Sequence Kernel Association Test (SKAT [29]) to test for the aggregate effect of multiple low-frequency variants within a gene on LOAD. Each study adjusted for age, sex, and for those principal components associated with LOAD (see **Supporting methods in S1 Text**). The discovery analysis was not adjusted for *APOE* genotypes; however this important LOAD risk factor was considered in secondary analyses of top findings. Both tests were performed in R with the seqMeta package (http://cran.r-project.org/web/packages/seqMeta/) to meta-analyze study-specific scores and respective variances and covariances (see details in **Supporting methods in S1 Text**) [28]. In the single-variant analysis we included variants present in at least two studies, variants with a minor allele frequency (MAF) ≥ 0.5% or assuming that rare damaging alleles could be prevalent predominantly in cases we also included variants with minor allele count (MAC) ≥ 5 in cases. This resulted in 52,026 single-variant tests. In the SKAT analysis we grouped the variants by genes using the start and stop positions as annotated by dbNSFP v2.0 [55] to define gene boundaries. We included only variants of MAF < 5% in the combined sample, and further annotated as missense, stop-gain, stop-loss, or splice-site variants. Then we required that genes have at least two such variants as well as at least two studies having a polymorphic variant. This resulted in 11,303 SKAT tests. After filtering, the Bonferroni correction thresholds that accounted for multiple testing were 9.6x10^-7^ for single-variant tests and 4.4x10^-6^ for SKAT. Two analysts independently performed the meta-analysis, leading to identical results.

#### Statistical methods in follow-up analysis of *TM2D3* in AGES

We used a score test and Fisher’s exact test for the follow-up analysis based on an independent Icelandic AGES sample (AGES-followup). We report (**Table 2**) estimates of the ORs based on fitting the full model (AD ~ age + sex + *TM2D3*) instead of the one-step approximation used in seqMeta (see **Supporting Methods in S1 Text** for details). For the AGES-discovery cohort we similarly update and report estimates of the ORs (**Table 2**) based on fitting the full model (**S1A Table**, **S2 Table**). In the AGES study, we also used a mixed-model to account for potential confounding effect relatedness might have on the association of P155L in *TM2D3* with LOAD (**Supporting results in S1 Text**). Cox regression was used to test for association of P155L with age-at-onset in the AGES data after adjusting for sex and *APOE* genotype. Individuals with LOAD diagnosis at the baseline visit were excluded so only at-risk individuals were included in the analysis. As detailed in the **Supporting methods in S1 Text**, the survival analysis accounted for left-truncation (i.e. follow-up begins after 65 years) and right-censoring (i.e. censoring that happens if a participant is lost to follow-up before having an event).

### Drosophila

Detailed methods for our *Drosophila* experiments can be found in the **Supporting methods in S1 Text**. Briefly, the rescue activity of *amx*, *TM2D3*, or *TM2D3*^*P155L*^ genomic constructs was examined in female flies of the genotype *amx*^*1*^*/Df(1)Exel9049; {Rescue Trangene}/+* or *amx*^*1*^*/Df(1)Exel9049; {Rescue Trangene}* when crossed to *amx*^*1*^ males (with or without the *{Rescue Transgene}*)[43]. To visualize the developing nervous system, resulting embryos were stained with anti-Hrp (1:1000) [56], a neuronal membrane marker and anti-ELAV (1:100) [57], a neuronal nuclear marker. For egg hatching, adults were allowed to lay eggs for 5 hours on grape juice agar plates, and larvae were counted 24 hours later.

## Supporting Information

S1 Text Supporting results and methods. Four supporting results sections and seven supporting methods sections, and sections with collaborators, and detailed funding information.
(PDF)

S1 Fig Quantile-quantile and cluster plots. Top row Quantile-quantile plots for the exome-wide discovery meta-analysis. Known genes are in orange. The genomic control coefficient (λ_GC_) is reported. Middle panel: Variants near *APOE* excluded Bottom row Cluster plots for SNPs in *SKAP2* and *TM2D3* demonstrate appropriate calling of the rare variant genotypes.
(PDF)

S2 Fig Schematic of *TM2D3* transcripts. A schematic of *TM2D3* transcripts. Ensembl identifiers (amino acid change due to rs13970957 in parenthesis) are marked by the schematic of each protein-coding transcripts. Bold text highlights transcripts that are also in RefSeq. The exon that contains rs13970957 is marked with an arrow. The schematic was retrieved 27 May 2016 from the Ensembl browser.
(PDF)

S3 Fig Expression of *TM2D3* in GTEx samples. Overall gene expression in various tissues, brain tissues are in yellow. TPM (“transcripts per million”) estimated using RSEM [58] on the GTEx data [37] (retrieved 26 May 2016 from UCSC Xena browser).
(PDF)

S4 Fig Expression of *TM2D3* protein-coding transcripts in GTEx tissues. Expression of protein-coding transcripts in all tissues (excluding blood), all brain tissues, and hippocampus only. TPM (“transcripts per million) estimated using kallisto [59] on the GTEx data [37] (retrieved 26 May 2016 from UCSC Xena browser). Transcript ENST00000559107 was not included in the plots because it is very lowly expressed and visualization was better without it.
(PDF)

S5 Fig Experimental crossing scheme for rescue experiments. The general crossing scheme is shown for the egg hatching rescue, quantified in Fig 2D. Due to the lethality of *Df(1)Exel9049/Y* hemizygous male progeny, complete rescue of the *amx* neurogenic phenotype is expected to lead to a maximum of ~75% egg hatching.
(TIFF)

S6 Fig Neurogenic phenotypes in developing flies. *TM2D3[+]* but not *TM2D3[P155L]* can suppress the neurogenic phenotype in the developing fly embryonic peripheral nervous system (PNS). (A-B) Maternal effect neurogenic defects in *amx* mutants can also be seen in the PNS. Compared to control embryos (A), maternal *amx* mutant embryos (B) show increased number of Sensory Organ Precursor cells (SOPs), labeled by Senseless (Sens, red), due to defective lateral inhibition. Embryos are counterstained with DAPI (blue). (C-F) PNS phenotypes in embryos laid by *amx* mutant females with or without *amx[+]*, *TM2D3[+]*, and *TM2D3[P155L]* genomic rescue constructs. Thoracic segments (T1, T2, T3) of stage 11 embryos are shown. *TM2D3[+]* can rescue the lateral inhibition defects in SOPs in some (Compare C, D and E) but not all embryos (E’). In contrast, all embryos from *amx* mutant females with *TM2D3[P155L]* exhibit neurogenic PNS phenotypes (F, F’). Scale bars = 100μm for A-B, = 50μm for C-F.
(TIFF)

S1 Table Results of exome-wide discovery analysis. Top results of exome-wide single variant (**A**) and SKAT (**B**) discovery analysis. Complete results areavailable in dbGAP (accession phs000930). A: Direction = AGES-CHS-FHS-RS, Alleles = non-coding/coding, AF displayed as percentage (%). B: cumAF = cumulative allele frequency displayed as percentage (%), N_SNPs = number of SNPs in test.
(PDF)

S2 Table Variant- and study-specific break-down of *TM2D3*, *SKAP2*, and *APOE* results. (PDF)

S3 Table Allele frequency of P155L in populations of European ancestry. (PDF)
