# Correction: Rare Functional Variant in *TM2D3* is Associated with Late-Onset Alzheimer's Disease

**DOI:** 10.1371/journal.pgen.1006456

**Published:** 2016-11-28

**Authors:** The PLOS Genetics Staff

The equal contributions of the last six authors are displayed incorrectly. The equal contributions for these authors should be stated as follows: “EB, BMP, SS, JMS, VG and CMvD contributed equally to this work.” The publisher apologizes for the error.

Two affiliations for the 53^rd^ author have been omitted. Joshua M. Shulman is also affiliated with: Department of Neurology, Baylor College of Medicine, Houston, Texas, United States of America, and Department of Neuroscience, Baylor College of Medicine, Houston, Texas, United States of America.
