# Supplementary material for: Rare Functional Variant in TM2D3 is Associated with Late-Onset Alzheimer's Disease
Source: PLoS Genet. 2016 Oct 20;12(10):e1006327. doi: 10.1371/journal.pgen.1006327 (PMC5072721; doi:10.1371/journal.pgen.1006327)
Supplement: S1 Text — Four supporting results sections and seven supporting methods sections, and sections with collaborators, and detailed funding information. (PDF) [file pgen.1006327.s001.pdf]

Table of Contents

Supporting results 2
Robustness of the AGES-discovery *TM2D3* result 2
TM2D3 haplotypes with rare variant 2
Effect of relatedness and substructure on *TM2D3* association 3
Competing risk due to death 3

Supporting methods 3
Details on the score test, one-step approximation to estimation of effect size, and seqMeta implementation 3
Statistical analysis 4
Population and pedigree substructure 4
Statistical methods in follow-up analysis of TM2D3 in AGES 5
Drosophila experiments 5
Stocks 5
Generation of amx/hTM2D3 genomic rescue constructs and transgenic flies 5
Embryo Immunofluorescence staining and imaging 6
Egg Hatching Assay 7
Sequence Alignment of TM2D3 homologs 7
CHARGE: Cohorts, LOAD diagnosis, and genotyping 7
Age Gene/Environment Susceptibility – Reykjavik study (AGES) 7
Cardiovascular Health Study (CHS) 9
Framingham Heart Study (FHS) 10
Rotterdam study (RS) 11
Genotyping in CHARGE cohorts 12
ADGC and GERAD: Cohorts, LOAD diagnosis, and genotyping 14
Alzheimer's Disease Genetics Consortium (ADGC) 14
Genetic and Environmental Research in Alzheimer's Disease (GERAD) 14
Cohorts that provided allele frequency estimates 17
Generation Scotland 17
GLACIER 17
DIABNORD 17
FIA3 17
Finrisk 18

Collaborators 19

Funding information 22

Supporting references 29

Supporting results

Robustness of the AGES-discovery *TM2D3* result

In the AGES-discovery sample we observed a score test p-value of 5.9×10^{-8} in a sample of 143 cases and 2374 controls. A p-value used to declare statistical significance was 9.6×10^{-7} (based on 52026 tests) and the control:case ratio is = 16.6. Under these circumstances: a small number of cases, an unbalanced sample (unequal numbers of cases and controls), and p-value that is less than an order of magnitude smaller than the significance cut-off, the score test could be anticonservative and the effect sizes could be over-estimated [1,2]. Before proceeding with further analysis, we therefore evaluated the robustness of the finding (1) by permutation testing ($p=6 \times 10^{-5}$ based on 10^6 replicates), (2) applying the Fisher's Exact test ($p=5.6 \times 10^{-4}$), and 3) applying bias-correction (bias-corrected OR is 4.5 [95% CI 2.4-9.0]) [2]. We concluded that the finding was robust, albeit possibly suffering from "winner's curse", and proceeded with the follow-up analysis.

TM2D3 haplotypes with rare variant

We used PHASE [3–5] (version 2.1) to estimate the haplotypes around the P155L variant. GWAS genotypes are available on the AGES-discovery cohort only, therefore the haplotype analysis applies to that cohort. The estimated haplotype of all individuals carrying the rare allele at P155L is TAAACG-**A-**GCCC for SNPs ranging from rs8043084 to rs716984. The probability of the estimated haplotype pair of each individual with the rare allele at P155L ranges from 0.998-1.000.

SNP	bp (build 38)	Location relative to *TM2D3* [1]	Allele
rs8043084	101601323	41kb 3' (intergenic)	T
rs7169834	101602444	39kb 3' (intergenic)	A
rs4965905	101606908	35kb 3' (intergenic)	A
rs12324658	101611170	31kb 3' (intergenic)	A
rs7168948	101617451	24kb 3' (intergenic)	C
rs477421	101622450	19kb 3' (intergenic)	G
**rs139709573 (P155L)**	**101646763**	**Exon of *TM2D3***	**A**
rs516667	101663828	Intron of *TARSL2*	G
rs2053701	101665206	Intron of *TARSL2*	C
rs12440512	101692267	Intron of *TARSL2*	C
rs8038261	101695258	Intron of *TARSL2*	C

[1] Relative to RefSeq as retrieved from Haploreg v4.1.

Effect of relatedness and substructure on *TM2D3* association

Adjusting for known kinship (pedigree structure is partially available for the AGES participants) using a mixed-model suggests that cryptic relatedness does not affect the significance of the association ($p_{discovery}$= 8.4x10^{-7}, $p_{followup}$= 0.0019, p_{pooled}=1.3x10^{-8}). The results were similarly robust to adjustment for kinship estimated empirically from GWAS genotypes, available on the discovery cohort, the significance is not affected ($p_{discovery}$=8.4x10^{-7}).

Competing risk due to death

To check whether there is evidence for bias due to competing risk of death, we used Cox regression to test whether *TM2D3* P155L was associated with mortality. This analysis did not yield evidence for association of *TM2D3* P155L with mortality (HR=1.4, 95% CI 0.9-2.1, p=0.1).

Supporting methods

Details on the score test, one-step approximation to estimation of effect size, and seqMeta implementation

In the discovery phase of our analysis (results in **Tables S2, S3, S4**) we used the meta-analysis implementation of a score test and set-based tests from the seqMeta R package. The seqMeta R package, like many other packages for meta-analysis of rare-variant associations, uses a one-step approximation to estimate effect sizes. The steps in seqMeta are as follows:

Single-variant analysis:

1) Each cohort fits a null model: phenotype ~ covariates
2) Each cohort calculates the residuals from the null model: residuals0
3) Each cohort calculates the score for each SNP: score_study = sum[residuals0 * genotype]
4) Each cohort calculates the score variance of the genotypes: var_study = sum(genotype-mean(genotype))^2
5) Each cohort shares: score_study, var_study, and MAF
6) The meta-analysis mscore = sum(score_study)
7) The meta-analysis score variance is mvar = sum(var_study)
8) The score test statistic is calculated as mscore^2/mvar which is compared to a X^2 distribution with 1 degree of freedom to get a p-value
9) Approximate effect size is estimated as beta = mscore/mvar
10) Standard error of beta is estimated as SE = 1/sqrt(mvar)

The effect sizes estimated this way are one-step approximation because we only fit the null regression model once without the genotypes.

Set-based (SKAT) analysis:

1) Each cohort fits a null model: Y ~ covariates

2) Each cohort calculate the residuals from the null model: residuals0
3) Each cohort calculates the score for each SNP: score_study = sum[residuals0 * genotype]
4) Each cohort calculates the covariance of the genotypes: cov_study
5) Each cohort shares the score_study, cov_study, and MAF
6) The meta-analysis mscore = sum(score_study)
7) The meta-analysis covariance is mcov = sum(cov_study)
8) The meta-analysis SKAT statistic is calculated as
9) Q = sum_{j=1…m} [w_j * beta_j / SE_beta_j^2, where, m=number of snps, p_j allele-frequence of SNP j based on the whole sample (aggregate allele frequency), w_j = 25*(1-p_j)^24, which is the "Wu weight" recommended to be used in SKAT for SNP j, beta_j and SE_beta_j are the meta-analysis beta and SE_beta for SNP j.
10) Q has an approximate distribution that is a linear combination of X^2, with the linear combination estimated from the genotype covariance matrix.

For both the single-variant and SKAT analysis steps 1-4 are done with the prepScores function of the seqMeta package. Then in the single-variant meta-analysis the singlesnpMeta function is used and in the SKAT analysis the skatMeta function is used to achieve steps 6-10.

Statistical analysis

Population and pedigree substructure

While logistic regression-based score tests can appropriately model dependence among observations (i.e. analysis of relatedness), similar methods have not yet been developed for multivariate tests such as SKAT. Meta-analyzing p-values would be possible but is typically much less powerful than meta-analysis of scores and respective variances (Cupples et al., 2012. Meta-analysis of a rare-variant association test. Unpublished technical report). Therefore, for the family-based cohort, FHS, we generated an unrelated subset of individuals to include in the analysis. This resulted in a loss of 7 cases and 590 controls. Because only 7 cases were lost and the control:case ratio was > 5 this step is unlikely to dramatically affect power of association analyses.

Each of the three randomly-ascertained population-based studies also investigated the degree of substructure and relatedness in their sample and made appropriate adjustments by: 1) removing one individual (preferably by case status) from a pair of individuals who appear closely related as empirically estimated from GWAS data and/or 2) testing for association of at least the top three principal components for population substructure (as estimated from GWAS data) with AD and adjusting for the ones that were associated with AD. We chose against adjustment for a pre-specified number of PCs in the discovery analysis because 1) the confounding due to rare and comment variants can be very different in structured populations and 2) methods that correct for population substructure in analysis of common variants (e.g. linear combination of PC

covariates), may not effectively control inflation in rare variant studies and can reduce power [6].

Statistical methods in follow-up analysis of TM2D3 in AGES

Survival and age-at-onset analysis in AGES – We performed survival analysis for the assocation of *TM2D3* P155L with age-at-onset for LOAD. Prevalent events as diagnosed at AGES-1 baseline visit were excluded so only at-risk individuals were included in the analysis, but o.w. the survival analysis included the same individuals as the previous association analysis. Left-truncation (i.e. follow-up begins after 65 years) and right-censoring (i.e. censoring that happens if a participant is lost to follow-up before having an event) were taken into account in the survival analysis. For the AGES-discovery cohort follow-up was set to the date of their AGES-2 visit (mean 5 years, range 3 to 8 years) or for those who did not attend AGES-2 visit follow-up was until the date of death or 6 years from the participant's AGES-1 exam date, whichever came first. For the AGES-followup cohort additional follow-up among individuals in nursing homes had become available. Therefore, for this sample, the follow-up time was set until last cognitive assessment (in nursing home or AGES-2), death, or until March 18 2014 (the date nursing home data were retrieved). The Kaplan-Meier estimator was used to estimate the survival function. Survival analysis stratified by sex and cohort and adjusted for *APOE*-ε4 was performed. The statistical significance for age at onset was assessed with a Wald test based on a Cox proportional hazard model, after assessing the proportional hazard assumption. Two-sided tests were implemented. The R package survival (version 2.36-14) was used.

Drosophila experiments

Stocks

The following stocks were obtained from Bloomington Drosophila Stock Center (BDSC) and used in this study:

amx^1 lz^g v^1/C(1)DX, y^1, f^1 (BDSC #10) [7]

Df(1)Exel9049 w1118/Binsinscy (BDSC #7770) [8]

y^1 M{vas-int.Dm}ZH-2A w; PBac{y[+]-attP-3B}VK00037* (BDSC #24872) [9]

All stocks were maintained on standard fly food at room temperature. All crosses were performed at 25 °C.

Generation of amx/hTM2D3 genomic rescue constructs and transgenic flies

For the *amx* genomic rescue construct, a 3,325 bp fragment corresponding to location X:9,245,044..9,248,369 [*Drosophila melanogaster* genome release 6 (Flybase: FB2015_03)] was cloned from P[acman] clone ch322-146A15 [10] using Xho I and Xba I restriction sites incorporated into the primers. The genomic DNA was PCR amplified via primers

ctctctCTCGAGGTTATGTTGCCTACATTTTGGTGCTCAC (Forward) and cttctTCTAGAGCGTCGCATCGTCAGTGAGGC (reverse) and cloned into the pattB vector [11]. For the human *TM2D3* knock-in rescue construct, the coding sequence of Amx within the *amx* genomic rescue construct was replaced precisely with the *TM2D3* variant 1 coding sequence cloned from the cDNA clone NM_078474 (Origene) flanked by BsaI restriction sites using primers gcggcGCTAGCGGTCTCtCAAAatggcgggaggggtgctccc (forward) and AGAGAGTCTAGAAGAGAGGTCTCGTAAACTAAATGTACAAAGAGCCATCTGCTGG (reverse) and assembled with the upstream region (ending prior to ATG) and the downstream region (beginning after the stop codon) from the *amx* genomic rescue construct using golden gate cloning [12,13]. For the *hTM2D3* P155L variant rescue construct, a single nucleotide change was introduced into the *hTM2D3* rescue construct via site directed mutagenesis by PCR using the TM2D3 Knock-In genomic rescue plasmid as a template with primers cctgtcctcggcagcgctaccttgccaactgcacggtgcggg and CCCGCACCGTGCAGTTGGCAAGGTAGCGCTGCCGAGGACAGG followed by DpnI (New England Biolabs) digestion. All constructs were verified using Sanger sequencing. Rescue construct containing the wild-type *amx* (amx[+]), wild-type *hTM2D3* (hTM2D3[+]) and P155L *hTM2D3* (hTM2D3[P155L]) coding sequence and *attB* site was injected into the 2nd chromosome *attP* docking site (VK37) via ϕC31 transgenesis [9,11]. Transgenic events were selected based on eye color (w^+) and the chromosomes were balanced over *SM6a*. These transgenes were crossed to 1st-2nd chromosome double balanced amx^1 lz^g v^1 flies to obtain the strains necessary for the rescue experiments.

Embryo Immunofluorescence staining and imaging

Fly crosses and egg collection: amx^1 lz^g v^1 males with or without the rescue transgene on the 2nd chromosome (*- ; amx[+]; hTM2D3[+]; hTM2D3[P155L]*) were crossed with *Df(1)Exel4049/Binsinscy* virgin females. In the next generation, amx^1 lz^g v^1*/Df(1)Exel4049; (Rescue Transgene)/+* virgin females were collected and crossed to amx^1 lz^g v^1 males. Fly crosses were allowed to lay eggs overnight (~16 hours) on a grape juice plate. Eggs/embryos laid on the plate were collected using standard procedures. Embryos were fixed and stained using standard procedures. In brief, embryos were washed in water, dechorionaed in 66% bleach solution for 4 minutes, and fixed in 3.7% formaldehyde/PBS/n-Heptane solution for 20 minutes. The fixed embryos were preserved in 100% methanol in -20°C till use. Embryos were rehydrated/permeabilized in 0.05% Triton-X in Phosphate Buffered Saline (PBST), and incubated with primary antibodies overnight. Antibodies used are anti-Hrp (1:1000, Rabbit polyclonal [14]), and anti-ELAV (Embryonic Lethal Abnormal Vision, 1:100, rat monoclonal (7E8A10) [15]). After additional washes, the embryos were incubated with secondary antibodies (anti-rat IgG Alexa Fluor 647, 1:200 anti-Rabbit IgG Alexa Fluor 488, 1:200 (Jackson ImmunoResearch)) and DAPI (1:100) for 1 hour at room temperature.

Following the final washes with PBST, Embryos were mounted with Vectashield onto a glass slide.
Microscopy: Stained Embryos were Imaged using LSM710, LSM880 or Apotome.2 system (Zeiss). Images were processed using ZEN software (Zeiss).

Egg Hatching Assay

Crosses (*amx*^{1} *lz*^{g} *v*^{1}*/Df(1)Exel4049; {Rescue Transgene}* virgin females x *amx*^{1} *lz*^{g} *v*^{1} males; *{Rescue Transgene}*) were set in plastic bottles and flies were allowed to lay eggs on grape juice plate with some yeast paste for 5 hours. All embryos from this cross will be homozygous for the *Rescue Transgene (amx[+]; hTM2D3[+]; hTM2D3[P155L]*). Embryos laid between this period were collected using a fine brush, washed in water, and dechorinated with 66% bleach for 4 minutes. Dechorinated embryos were washed again, and put into a 24-well cell culture plate (BD Falcon) well that contains 500 uL of 1x PBS. Embryos will float at the top, and a photograph of each well was taken at the beginning and at the end of a 24 hour incubation period at 25°C. The number of larva that hatched was counted and the egg hatching rate (%) was calculated by dividing this number by the number of eggs being laid. The process was continued for 5 days.

Sequence Alignment of TM2D3 homologs

Protein sequence alignment of TM2D3 homologs were performed using Clustal X (2.1) using the standard parameters (http://www.clustal.org/clustal2/). Homologs of TM2D3 in human, mouse (Mus musculus), zebrafish (*Danio rerio*), fly (*Drosophila melanogaster*), and worm (*Caenorhabditis elegans*) were identified via HCOP (http://www.genenames.org/cgi-bin/hcop). The respective gene symbol in each species are: *TM2D3* (human), *Tm2d3* (mouse), *tm2d3* (zebrafish), *amx* (fly), and *C41D11.9* (worm).

CHARGE: Cohorts, LOAD diagnosis, and genotyping

Age Gene/Environment Susceptibility – Reykjavik study (AGES)

The AGES study has been described previously [16].The study was initiated in 2002 to examine genetic susceptibility and gene/environment interactions related to disease and disability in old age. The AGES study is comprised of 5764 individuals drawn from the Reykjavik Study, a population-based cohort comprised of individuals born between 1907 and 1935 and followed since 1967 by the Icelandic Heart Association. 3219 individuals chosen randomly among 5307 AGES individuals with ‘mid-life’ data available from the Reykjavik Study were genotyped on a genome-wide association (GWA) array. 2983 were further genotyped for the EC.

AGES-discovery cohort – Individuals genotyped for the EC represent the discovery sample ('AGES-discovery') in the analysis (**Table 1**). None of the top 3 principal components (PCs) as derived from GWA data were found associated with LOAD and thus no PCs were used as covariates and only age and sex were included as covariates. Age was coded in years where the age of cases was the age at the visit where LOAD was first diagnosed and the age of controls was the age at the last visit individual was still free of LOAD pathology.
AGES-followup cohort – After discovery phase AGES participates who were not genotyped for the EC ('AGES-followup') were genotyped for the *TM2D3* variant (**Table 2**). GWA data were not available. Age was coded the same way as in the AGES-discovery cohort
Diagnosis of LOAD in AGES cohorts – Individuals from both cohorts were assessed at two visits (AGES-1 and AGES-2) to the study center with approximately 5 years between them. The Folstein Mini Mental State Examination (MMSE) and the Digit Symbol Substitution Test (DSST) were administered to all participants and persons who scored below a pre-determined threshold on these tests (≤23 on the MMSE or ≤17 on the DSST) were administered a second, diagnostic test battery. Based on performance on the Trails B and the Rey Auditory Verbal Learning test (RAVLT), a subset of these individuals with a RAVLT score ≤18 or Trails B score ≥8 (ratio of time taken for Trails B/Trails A corrected for the number correct) went on to a third step, which included a neurological examination and a structured informant interview about medical history and social, cognitive, and daily functioning. MRI was acquired as a part of the core study protocol. A panel that included a geriatrician, neurologist, neuropsychologist, and neuroradiologist reached a consensus diagnosis of dementia based on the *Diagnostic and Statistical Manual of Mental Disorders,* Fourth Edition (DSM-IV) guidelines [17]. There were 319 cases of dementia diagnosed in the first 5764 AGES participants and of these 123 also had genotyping and brain MRI. International diagnostic guidelines, including the National Institute of Neurological and Communicative Disorders and Stroke–Alzheimer Disease and Related Disorders Association (NINCDS- ADRDA) criteria for probable and possible Alzheimer Disease and the Alzheimer's Disease Diagnosis and Treatment Center's (ADDTC) State of California criteria for probable and possible vascular dementia (VaD) with or without AD, were followed. The AGES study identified 3 subtypes: possible/probable AD without VaD (included in analysis), mixed AD (cases that met criteria for both AD and VaD, included in analysis), and, possible/probable VaD or other dementia without AD (excluded from analysis). 3316 individuals participated in the follow-up visit (AGES-2) and were examined using the same protocol as used during the AGES-1 visit for diagnosis of dementia and AD. For the AGES-followup cohort additional follow-up was available after AGES-1 on 966 AGES participants in nursing homes (or home care) who had been systematically followed using the Resident Assessment Instrument (RAI) [18]. This allowed for a more thorough follow-up and less misclassification of cases as controls. The RAI is an

internationally validated instrument for systematic assessment of nursing home residents [19–21]. The majority of Icelanders suffering cognitive decline undergo assessments at the Memory Clinic at the National University Hospital of Iceland, where LOAD is diagnosed according to the international NINCDS-ADRDA criteria for definite, probable, or possible AD [22]. The LOAD diagnosis from the Memory Clinic is subsequently documented on the Minimum Data Set 2.0 of the RAI. In both cohorts, controls were those still free of dementia and mild cognitive impairment at last assessment.
Study approval – The AGES study was approved by the Icelandic National Bioethics Committee (VSN 00-063), and by the National Institute on Aging Intramural Institutional Review Board. Informed consent was obtained from all participants.

Cardiovascular Health Study (CHS)

The CHS is a population-based cohort study of risk factors for coronary heart disease and stroke in adults ≥65 years conducted across four field centers [23]. The original predominantly Caucasian cohort of 5201 persons was recruited in 1989-1990 from random samples of the Medicare eligibility lists; subsequently, an additional predominantly African-American cohort of 687 persons was enrolled for a total sample of 5888. Blood samples were drawn on all participants at their baseline examination; DNA was extracted from blood from participants who donated DNA samples for storage and provided informed consent for participation in DNA studies (~95% of all CHS participants). Although CHS is a population-based sample we empirically estimated cryptic relatedness based on genotypes of a LD-pruned set of common EC variants. For this we used PLINK v1.07 [24] (http://pngu.mgh.harvard.edu/purcell/plink/). We identified clusters of individuals with 'PI_HAT' > 0.15 or 'Z0' < 0.4 ('PI_HAT' is the empirical estimate of twice the kinship coefficient and Z0 is the empirical estimate of the probability of sharing zero alleles identical by decent). Among these clusters, we kept only one individual for analysis, giving preference to cases over controls. Covariates in the models were age in years, sex, and field center. Age was the age at LOAD diagnosis for cases or the age at last follow-up evaluation for controls. PCs were not associated with LOAD and therefore not included in the final model.
Diagnosis of LOAD in CHS – The AD sample for CHS included all prevalent cases identified in 1992 and incident events identified between 1992 and December 2006. Briefly, persons were examined annually from enrollment to 1999, and the examination included a 30 minutes screening cognitive battery [25]. In 1992-94 and again, in 1997-99, participants were invited to undergo brain MRI and detailed cognitive and neurological assessment as part of the CHS Cognition Study [25]. Persons with prevalent dementia were identified, and all others were followed until 1999 for the development of incident dementia and AD. Since then, CHS participants at the Maryland and Pennsylvania centers have remained under ongoing dementia surveillance [26]. Beginning in 1988/89, all participants completed the Modified Mini-Mental State Examination (3MSE) and

the DSST at their annual visits, and the Benton Visual Retention Test (BVRT) from 1994 to 1998. The Telephone Interview for Cognitive Status (TICS) was used when participants did not come to the clinic. Further information on cognition was obtained from proxies using the Informant Questionnaire for Cognitive Decline in the Elderly (IQCODE), and the dementia questionnaire (DQ). Symptoms of depression were measured with the modified version of the Center for Epidemiology Studies Depression Scale (CES-D). In 1991-94, 3608 participants had an MRI of the brain and this was repeated in 1997-98. The CHS staff also obtained information from participants and next-of-kin regarding vision and hearing, the circumstances of the illness, history of dementia, functional status, pharmaceutical drug use, and alcohol consumption. Data on instrumental activities of daily living (IADL), and activities of daily living (ADL) were also collected. Persons suspected to have cognitive impairment based on the screening tests listed above underwent a neuropsychological and a neurological evaluation. The neuropsychological battery included the following tests: the American version of the National Reading test (AMNART), Raven's Coloured Progressive Matrices, California Verbal Learning Test (CVLT), a modified Rey-Osterreith figure, the Boston Naming test, the Verbal fluency test, the Block design test, the Trails A and B tests, the Baddeley & Papagno Divided Attention Task, the Stroop, Digit Span and Grooved Pegboard Tests. The results of the neuropsychological battery were classified as normal or abnormal (>1.5 standard deviations below individuals of comparable age and education) based on normative data collected from a sample of 250 unimpaired subjects. The neurological exam included a brief mental status examination, as well as a complete examination of other systems. The examiner also completed the Unified Parkinson's Disease Rating Scale (UPDRS) and the Hachinski Ischemic Scale. After completing the neurological exam, the neurologist classified the participant as normal, having mild cognitive impairment (MCI), or dementia. International diagnostic guidelines, including the NINCDS-ADRDA criteria for probable and possible AD and the ADDTC's State of California criteria for probable and possible vascular dementia (VaD) with or without AD, were followed. CHS identified 3 subtypes: possible/probable AD without VaD (categorized as pure AD, included in analysis) and mixed AD (for cases that met criteria for both AD and VaD, included in analysis), and, possible/probable VaD without AD (excluded from current study).

Framingham Heart Study (FHS)

The FHS is a three generational prospective cohort that has been described in detail previously [27–29]. Individuals were initially recruited in 1948 in Framingham, MA, USA to evaluate cardiovascular disease risk factors. The second-generation cohort (5,124 offspring of the original cohort) was recruited between 1971 and 1975. The third-generation cohort (4095 grandchildren of the original cohort) was collected between 2002 and 2005. 6946 European-American individuals were genotyped using the EC. Participants ≤60 years at the time of

blood draw for DNA extraction were excluded prior to analysis. Because the statistical tests used did not account for family structure, we excluded related participants. Using genome-wide identity-by-descent, we first identified 7 pairs of related cases, and excluded the younger of the two in each pair, or the one with the most missing data. We then excluded 151 controls who were related to cases, and finally, we excluded 439 controls related to other controls, applying the same age/missing data rule as for related cases. Covariates used were age in years and sex, where age was the age at LOAD diagnosis for cases or the age at last follow-up evaluation for controls.

Diagnosis of LOAD in FHS – FHS participants were screened at each biennial examination for possible cognitive decline through a number of mechanisms, including measures of the Folstein Mini-Mental Status Examination (MMSE) [30], referral by FHS staff and physicians at regular clinic exams, by self, family or primary care physician, referral following health updates or ancillary studies by other FHS working groups, and referral from neuropsychological testing included in dedicated project. Participants "flagged" as being at risk for developing dementia underwent complete neuropsychological evaluation. If the neuropsychological testing or neurological evaluation suggested a decline in cognitive function, and other sources of data could not clarify if the person had MCI or AD, we administered a structured family interview. We then determined whether each person fulfilled criteria for a diagnosis of dementia, the probable date of onset, and type of dementia at a consensus review conducted by a panel comprising at least one behavioral neurologist and one neuropsychologist. Participants with dementia met criteria outlined in the Fourth edition of the Diagnostic and Statistical Manual of Mental Disorders (DSM-IV) criteria [17], and were required to have symptoms for at least 6 months. Participants with AD met NINCDS-ADRDA criteria for definite, probable, or possible AD [22].

Rotterdam study (RS)

The RS is an ongoing prospective population-based cohort study, focused on chronic disabling conditions of the elderly [31]. The study comprises an outbred ethnically homogenous population of Dutch Caucasian origin. The rationale of the study has been described in detail elsewhere [31]. In summary, 7983 men and women aged 55 years or older, living in Ommoord, a suburb of Rotterdam, the Netherlands, were invited to participate. 3163 individuals were genotyped for the EC. 1764 individuals have exome sequencing data and 820 of those also have EC data. In the RS there are some small families due to inclusion of parents as well as children living both in Ommoord. From pairs of subjects with empirical IBD>0.4 one was excluded, with a preference of keeping cases. This excluded 30 cases and 120 controls. None of first 10 PCs related to AD in any of the samples and thus only age and sex were included as covariates. Age was coded in years for age of onset for cases and age at censoring or age at last screening for controls.

Diagnosis of LOAD in RS – In the RS participants were screened for prevalent dementia in 1990-93 using a three-stage process; those free of dementia remained under surveillance for incident dementia, a determination made using records linkage and assessment at three subsequent re-examinations. We included all prevalent cases and all incident events up to January 1st 2011. Screening was done with the Folstein Mini-Mental Status Examination (MMSE) [30] and the Geriatric Mental Schedule (GMS) [32] organic level for all persons. Screen-positives (MMSE < 26 or GMS organic level > 0) underwent the CAMDEX [33]. Persons who were suspected of having dementia underwent more extensive neuropsychological testing. When available, imaging data were used. In addition, all participants have been continuously monitored for major events (including dementia) through automated linkage of the study database with digitized medical records from general practitioners, the Regional Institute for Outpatient Mental Health Care and the municipality. In addition physician files from nursing homes and general practitioner records of participants who moved out of the Ommoord district were reviewed twice a year. For suspected dementia events, additional information (including neuroimaging) was obtained from hospital records and research physicians discussed available information with a neurologist experienced in dementia diagnosis and research to verify all diagnoses. Dementia was diagnosed in accordance with internationally accepted criteria for dementia (Diagnostic and Statistical Manual of Mental Disorders, Revised Third Edition, DSM-III-R [34]), and AD using the NINCDS-ADRDA criteria for possible, probable and definite AD [22]. The National Institute of Neurological Disorders and Stroke–Association Internationale pour la Recherche et l'Enseignement en Neurosciences (NINDSAIREN) criteria were used to diagnose vascular dementia. The final diagnosis was determined by a panel of a neurologist, neurophysiologist, and research physician and the diagnoses of AD and VaD were not mutually exclusive.
Study approval – The Rotterdam Study has been approved by the Medical Ethics Committee of the Erasmus MC and by the Ministry of Health, Welfare and Sport of the Netherlands implementing the Wet Bevolkingsonderzoek: ERGO (Population Studies Act: Rotterdam Study). All participants provided written informed consent to participate in the study and to obtain information from their treating physicians.

Genotyping in CHARGE cohorts

ExomeChip genotyping and quality control – All four CHARGE cohorts were genotyped for the HumanExome BeadChip v1.0 from Illumina (Illumina, Inc., San Diego, CA, USA). To increase the quality of the rare variant genotype calls, the genotypes for all four studies were jointly called with 62,266 samples from 11 studies at the University of Texas HSC at Houston (UT Houston) [35]. Quality control (QC) procedures for the genotype data were done both centrally at UT Houston and at each study. The central QC procedures have been described previously [35]. Each study performed QC locally, which included at the

minimum: 1) Concordance checking with GWAS data and removal of problematic samples, 2) Removal of individuals with low genotype completion rate (<90%), 3) Removal of variants with low genotype call rate (<95%), 4) Removal of individuals with sex-mismatches, 5) Removal of one individual from duplicate pairs, 6) Removal of variants that deviated significantly form the expected Hardy-Weinberg Equilibrium proportions ($p<10^{-6}$).

Genotype success rate by cohort – There were a total of 247870 variants on the ExomeChip. After removing 831 duplicate SNPs, 5574 non-autosomal SNPs, and 8581 variants excluded during the central calling at UT Houston 241465 remained. Of those 241465 variants 99.9%, 95.5%, 99.9%, 99.5% variants passed QC for AGES, CHS, FHS, and RS, respectively.

Annotation – DBNSFP v2.0 was used to annotate the variants [36].

Quality of the ExomeChip genotypes – The quality of the calling of the ExomeChip genotypes within CHARGE has been described previously [35]. We further investigated the quality of the genotypes of the SNPs in *SKAP2* and *TM2D3* by 1) visually confirming cluster plots (**Figure S1B**), 2) genotyping all individuals from the AGES-discovery who had the rare variant (rs139709573) at *TM2D3* (100% validated), and 3) checking concordance with exome sequencing for total of 820 samples that had both EC and ES data available in the RS (99.6% concordant).

Exome Sequencing in RS – Exomes of 1764 individuals from the RS-I population were sequenced using the Nimblegen SeqCap EZ V2 capture kit on an Illumina Hiseq2000 sequencer and the TrueSeq Version 3 protocol. The sequences reads were aligned to the human genome build 19 (hg19) using Burrows-Wheeler Aligner [37]. Subsequently, the aligned reads were processed further using Picard (http://picard.sourceforge.net), SAMtools [38] and Genome Analysis Toolkit (GATK) [39]. Genetic variants were called using Unified Genotyper Tool from GATK. Samples with low concordance to genotyping array (< 95%), low transition/transversion ratio (<2.3) and high heterozygote to homozygote ratio (>2.0) were removed from the data. The final dataset consisted of 903,316 SNVs in 1524 individuals, of which 820 were also genotyped for the EC. Variants called on both genotyping platforms were compared. In total 68,379 variants were both called on the exome chip and with exome sequencing. We matched the reference and alternate alleles and calculated the concordance rate for all variants. Pairs were the exome sequence genotype was missing were excluded (n=2,884,491) in the comparison. A total of 52,823,580 pairs of genotypes were tested and 99.563% were concordant, 0.364% discordant and 0.073% missing in the exome chip data. These results are comparable to previous results [35].

Coverage of the ExomeChip – The Illumina HumanExome Beadchip (“the exome array”) was designed based on variants discovered through sequencing of ~12,000 genomes or exomes (http://genome.sph.umich.edu/wiki/Exome_Chip_Design). The coverage of this exome chip has been evaluated in independent samples and is estimated to

capture ~78% of missense and splice-site variants of >0.1% allele frequency in individuals of European descent [40]

ADGC and GERAD: Cohorts, LOAD diagnosis, and genotyping

Alzheimer's Disease Genetics Consortium (ADGC)

The National Institute on Aging (NIA) Alzheimer Disease Centers (ADC) case-control sample, the University of Toronto/GlaxoSmithKline (also called Gen ADA) case-control sample, the Vanderbilt/Miami/Mt. Sinai case-control sample, the NIA-Late-Onset AD (NIA-LOAD) multiplex family-based sample, the National Cell Repository for Alzheimer's Disease (NCRAD) multiplex family-based sample, the Multi-Institutional Research in Alzheimer's Genetic Epidemiology (MIRAGE) family-based sample, and the Adult Changes in Thought (ACT) prospective cohort were described previously [41,42]. The Genetics Differences cohort is a population-based prevalent case-control study from the same population as the ACT study [43]. The Washington Heights Inwood Columbia Aging Project (WHICAP) sample is a multi-ethnic prospective cohort [44]; for this study, only Caucasians were genotyped. The Miami multiplex families and the National Institute on Mental Health multiplex families were as previously described [45–47]. The Cache County Study on Memory in Aging is a population-based study with four assessments of cognitive function since 1994 [48]. The Swedish cohorts are case-control studies recruited from neuropsychiatric clinics in Sweden, as described previously [49]. For the family-based sample, we genotyped a single affected subject from each kindred. All studies were approved by Institutional Review Boards (IRBs) for each study by the respective Universities involved in each study and the overall study was approved by the University of Pennsylvania IRB.

ADGC participants were genotyped using the Infinium HumanExome V1 Beadchip from Illumina. Genotyping for 8410 subjects was performed at NorthShore, 1990 subjects at the Hussman Institute for Human Genomics at the University of Miami, and 6166 subjects at Center for Applied Genomics at Children's Hospital of Philadelphia. Genotypes were initially called using the default clustering profile from Illumina and recalled using clustering profiles generated by Genentech using data from 30,000 samples.

Genetic and Environmental Research in Alzheimer's Disease (GERAD)

MRC genetic resource for late-onset AD (MRC LOAD): Samples were recruited by the MRC Genetic Resource for AD (Cardiff University; Institute of Psychiatry, London; Cambridge University and Trinity College Dublin). All AD cases met criteria for either probable (NINCDS-ADRDA5, DSM-IV) or definite (CERAD) AD. All individuals included in these analyses have provided informed consent to take part in genetic association studies. MRC Prion Unit: Patients were recruited via

tertiary specialist clinics at the National Hospital for Neurology and Neurosurgery, University College London Hospitals NHS Foundation Trust, London. Clinical diagnosis of AD was supported by participation in longitudinal research studies at University College London, however as these sample collections were acquired over two decades the comprehensive use of research diagnostic criteria cannot be confirmed and some samples were assigned based on clinical diagnoses only. Southampton: Subjects were aged between 50 and 100 years and screened using the MOCA tool with a MoCA score at baseline equal to or greater than 26 points. Signed informed consent was obtained by subject prior to the initiation of any study-specific procedure and defined inclusion and exclusion criteria were used. Kings College London: Subjects were assessed using the MMSE and diagnosed according to the criteria of the NINCDS-ADRDA. All patients had an age of onset greater than 60 years and controls were 60 years or older at time of recruitment. University of Nottingham: Informed consent was obtained for all samples, which was approved by the local Ethics Committee. Samples were histopathologically confirmed as definite disease (AD) or control using CERAD criteria. Queen's University, Belfast: Diagnosis was based on DSM IV and NINCDS ADRDA, as assessed by two clinicians. MMSE controls > 28/30, Age > 65 for cases/controls. Centro de Biología Molecular Severo Ochoa (CSIC-UAM): AD patients were clinically diagnosed based on NINCDS-ADRDA or DSM-IV criteria. Controls were ascertained by a mini-mental exam. All subjects gave their informed consent to participate in the study. University of Halle, Germany: Patients: All subjects' samples were collected at the University, Munich. The study was approved by the Munich University Ethics Committee and all participants provided informed written consent. Participants diagnosed with dementia associated with Alzheimer's disease fulfilled the criteria of probable Alzheimer´s disease, according to the NINCDS-ADRDA. Cognitive testing by neuropsychological evaluation was performed in all patients according to MMSE, CERAD battery, multiple choice vocabulary test and a variant of the trail making test. Controls: Healthy subjects were screened to exclude those with neuropsychiatric disorders according to defined exclusion criteria. University of Bonn: German AD patients sample from Bonn were recruited from the German Dementia Competence Network, the German study on Aging, Cognition, and Dementia in primary care patients (AgeCoDe) and the interdisciplinary Memory Clinic at the University Hospital of Bonn. All AD dementia patients included in the GWAS analysis fulfilled the NINCDS/ADRDA criteria for probable AD and were assessed by CERAD; the MMSE; the CDR, DSM-IV, ICD-10 (SIDAM), laboratory assessments, brain imaging or NINCDS/ADRDA criteria. Control samples comprised healthy elderly individuals from the AgeCoDe cohort with absence of any type of dementia and mild cognitive impairment diagnosis using SIDAM. The study was approved by all respective ethics committees and written informed consent was obtained. Universitari Mutua de Terrassa, Barcelona and University of Navarra, Pamplona Spain: All patients were assessed with MMSE and diagnosed according to the criteria of the National Institute of Neurological and

Communication Disorders and Stroke (about 10% of the controls underwent also an MMSE). Informed consent was obtained from all individuals or family representatives. Santa Lucia Foundation: For the enrollment of AD patents, the MMSE and the MDB were administered. Diagnosis was made according to the NINCDS-ADRDA criteria CDR score equal or higher than 1.0. Subjects were aged greater or equal to 56 years old (patients) and 55 years old (healthy controls). Brigham Young University and Utah State University: Case-control status was determined in the Cache County Study on Memory Health and Aging cohort who were aged 65 and older in a multi-stage dementia screening and assessment protocol utilising the Modified Mini-Mental State Exam-Revised (3MS-R) and other tests. Diagnoses of AD followed NINCDS-ADRDA criteria. Controls were identified as those who were diagnosed with no dementia (per clinical assessment) or whose cognitive test result was negative. All study procedures were approved by the Institutional Review Boards of Utah State, Duke and the Johns Hopkins University. Washington University: The Institutional Review Board at the Washington University School of Medicine in Saint Louis approved the study. Written informed consent was obtained from participants and their family members by the Clinical Core of at the Charles F. and Joanne Knight Alzheimer's Disease Research Center (Knight-ADRC) (Approval number 93-0006). Cases received a clinical diagnosis of AD dementia in accordance with standard criteria, and dementia severity was determined with the CDR. Controls underwent the same assessment but were cognitively normal. Hospital de la Sant Pau, Universitat Autònoma de Spain: Diagnosis of AD was established according to the NINDS-ADRDA guidelines. Control individuals were older than 60 years of age and had a neuropsychological evaluation in the normal range for age and education. The study was approved by the ethics committee at the centre. Saarland University: All patients underwent a thorough clinical and neuropsychological examination including the CERAD-NP test battery with the MMSE and a CDR rating. Further exams include physical and neurological examination and a brain scan (MRI). Diagnoses was performed according to the NINCDS-ADRDA criteria. Further information includes gender, current age, age of onset, which in this cohort was above 60 years. Informed consent was obtained from each participant and further approval by the ethics committee was also obtained. Participants with diagnoses other than AD were excluded in particular those with mixed dementias, FTLD and if the age of onset was below 60 years.

GERAD participants were genotyped using either the Illumina HumanExome V1.0 (995 cases, 3383 controls) or V1.1 (1127 cases, 1729 controls) Beadchips. Genotyping for all subjects was performed at Life & Brain GmbH, Bonn, Germany. Genotypes were initially called using the Illumina GenomeStudio software (default settings). Genotypes were recalled using zCall to improve rare variant calling [50]. Cluster plots for markers of interest were visually inspected.

Cohorts that provided allele frequency estimates

Generation Scotland

Generation Scotland is a Scottish family- and population-based adult cohort [51]. Generation Scotland received core support from the Chief Scientist Office of the Scottish Government Health Directorates [CZD/16/6] and the Scottish Funding Council [HR03006]. Ethical approval for the study was obtained from the Tayside Committee on Medical Research Ethics (on behalf of the National Health Service). Genotyping was funded by the UK Medical Research Council.

GLACIER

The Gene-Lifestyle interactions And Complex traits Involved in Elevated disease Risk (GLACIER) Study is nested within the Västerbotten Health Survey, which is part of the Northern Sweden Health and Disease Study, a population-based prospective cohort study from northern Sweden [52]. A total of 965 non-diabetic participants from the GLACIER Study had complete genotype data. Participants were genotyped with IlluminaHumanExomeBeadchip 12 v1.1. All participants provided informed consent. Ethics committee approval no. 2011-243-32M.

DIABNORD

The DIABNORD Study is nested within the Västerbotten Health Survey, which is part of the Northern Sweden Health and Disease Study, a population-based prospective cohort study from Northern Sweden [52]. Participants with incident type 2 diabetes were identified from the Diabetes Register in Northern Sweden (DiabNorth). A total of 928 participants with incident type 2 diabetes from the DIABNORD Study had complete genotype data. Participants were genotyped with IlluminaHumanExomeBeadchip 12 v1.1. All participants provided informed consent. Ethics committee approval no. 2011-243-32M.

FIA3

FIA3 is population-based study of myocardial infarction (MI) nested within the Northern Sweden Health and Disease Study (NSHDS), a population-based cohort study from northern Sweden, which consists of sub-cohorts: the Västerbotten Intervention Program (VIP) and the WHO's Multinational Monitoring of Trends and Determinants in Cardiovascular Disease (MONICA) Study in northern Sweden [52]. Both VIP and MONICA are health examination programs for cardiovascular disease (CVD) and diabetes. Cases are identified through the MONICA study in northern Sweden and its MI incidence registry. Study participant were genotyped with IlluminaHumanExomeBeadChip 12 v1.1. All participants provided informed consent. Ethics committee approval no. 2011-87-31M

Finrisk

For each Finrisk (1992, 1997, 2002) cohort [53], a representative random sample is selected from the 25 – 74 year old inhabitants in five regions of Finland. The survey includes a mailed questionnaire and a clinical examination where a blood sample is drawn. A total of 23,036 individuals participated in the cohorts, and gave written informed consent. The genotyped subset is a random sample that was genotyped using Illumina HumanCoreExome chip. The FINRISK data are deposited in the THL Biobank, which has been approved by the Coordinating Ethical Committee of the Helsinki University Hospital District (decision number 238/13/03/00/2014)

Collaborators

CHARGE: Ben A. Oostra, PhD; Peter J. Koudstaal, MD PhD; Myriam Fornage, PhD, Thomas H. Mosley, PhD; Jan Bressler, PhD

ADGC: Marilyn S. Albert, PhD; Roger L. Albin, MD; Liana G. Apostolova, MD; Steven E. Arnold, MD; Sanjay Asthana, MD; Craig S. Atwood, PhD; Clinton T. Baldwin, PhD; Michael M. Barmada, PhD; Lisa L. Barnes, PhD; Thomas G. Beach, MD PhD; James T. Becker, PhD; Gary W. Beecham, PhD; Duane Beekly, BS; Timothy W. Behrens, MD; David A. Bennett, MD; Eileen H. Bigio, MD; Thomas D. Bird, MD; Deborah Blacker, MD; Bradley F. Boeve, MD; James D. Bowen, MD; Adam Boxer, MD PhD; James R. Burke, MD PhD; Joseph D. Buxbaum, PhD; Nigel J. Cairns, PhD FRCPath; Laura B. Cantwell, MPH; Chuanhai Cao, PhD; Chris S. Carlson, PhD; Cynthia M. Carlsson, MD; Regina M. Carney, MD; Steven L. Carroll, MD PhD; Helena C. Chui, MD; David G. Clark, MD; Paul K. Crane, MD MPH; David H. Cribbs, PhD; Elizabeth A. Crocco, MD; Carlos Cruchaga, PhD; Charles DeCarli, MD; F. Yesim Demirci, MD; Malcolm Dick, PhD; Dennis W. Dickson, MD; Ranjan Duara, MD; Nilufer Ertekin-Taner, MD PhD; Kelley M. Faber, MS; Kenneth B. Fallon, MD; Martin R. Farlow, MD; Lindsay A. Farrer, PhD; Steven Ferris, PhD; Tatiana M. Foroud, PhD; Matthew P. Frosch, MD PhD; Douglas R. Galasko, MD; Marla Gearing, PhD; Daniel H. Geschwind, MD PhD; Bernardino Ghetti, MD; John R. Gilbert, PhD; Jonathan D. Glass, MD; Alison M. Goate, D.Phil; Neill R. Graff-Radford, MD; Robert R. Graham, PhD; Robert C. Green, MD MPH; John H. Growdon, MD; Jonathan L. Haines, PhD; Hakon Hakonarson, MD PhD; Ronald L. Hamilton, MD; Kara L. Hamilton-Nelson, MPH; Lindy E. Harrell, MD PhD; Elizabeth Head, PhD; Lawrence S. Honig, MD PhD; Ryan M. Huebinger, PhD; Christine M. Hulette, MD; Bradley T. Hyman, MD PhD; Gail P. Jarvik, MD PhD; Gregory A. Jicha, MD PhD; Lee-Way Jin, MD PhD; Gyungah Jun, PhD; M. Ilyas Kamboh, PhD; Anna Karydas, BA; John S.K. Kauwe, PhD; Jeffrey A. Kaye, MD; Ronald Kim, MD; Neil W. Kowall, MD; Joel H. Kramer, PsyD; Walter A. Kukull, PhD; Brian W. Kunkle, PHD MPH; Frank M. LaFerla, PhD; James J. Lah, MD PhD; Eric B. Larson, MD MPH; James B. Leverenz, MD; Allan I. Levey, MD PhD; Ge Li, MD PhD; Andrew P. Lieberman, MD PhD; Chiao-Feng Lin, PhD; Oscar L. Lopez, MD; Kathryn L. Lunetta, PhD; Constantine G. Lyketsos, MD MHS; Wendy J. Mack, PhD; Daniel C. Marson, JD PhD; Eden R. Martin, PhD; Frank Martiniuk, PhD; Deborah C. Mash, PhD; Eliezer Masliah, MD; Richard Mayeux, MD; Wayne C. McCormick, MD MPH; Susan M. McCurry, PhD; Andrew N. McDavid, BA; Ann C. McKee, MD; Marsel Mesulam, MD; Bruce L. Miller, MD; Carol A. Miller, MD; Joshua W. Miller, PhD; Thomas J. Montine, MD PhD; John C. Morris, MD; Shubhabrata Mukherjee, PhD; Jill R. Murrell, PhD; John M. Olichney, MD; Joseph E. Parisi, MD; Amanda Partch, MS; Henry L. Paulson, MDPhD; Margaret A. Pericak-Vance, PhD; William Perry, MPH; Elaine Peskind, MD; Ronald C. Petersen, MD PhD; Aimee Pierce, MD; Wayne W. Poon, PhD; Huntington Potter, PhD; Joseph

F. Quinn, MD; Ashok Raj, MD; Murray Raskind, MD; Eric M. Reiman, MD; Barry Reisberg, MD; Christiane Reitz, MD PhD; John M. Ringman, MD; Erik D. Roberson, MD PhD; Ekaterina Rogaeva, PhD; Howard J. Rosen, MD; Roger N. Rosenberg, MD; Mark A. Sager, MD; Mary Sano, PhD; Andrew J. Saykin, PsyD; Julie A. Schneider, MD; Lon S. Schneider, MD; William W. Seeley, MD; Amanda G. Smith, MD; Joshua A. Sonnen, MD; Salvatore Spina, MD; Peter St George-Hyslop, MD FRCP; Robert A. Stern, PhD; Rudolph E. Tanzi, PhD; Tricia A. Thornton-Wells, PhD; John Q. Trojanowski, MD PhD; Juan C. Troncoso, MD; Debby W. Tsuang, MD; Otto Valladares, MS; Vivianna M. Van Deerlin, MD PhD; Linda J. Van Eldik, PhD; Badri N. Vardarajan, MS; Harry V. Vinters, MD; Jean Paul Vonsattel, MD; Sandra Weintraub, PhD; Kathleen A. Welsh-Bohmer, PhD; Jennifer Williamson, MS; Sarah Wishnek, MPH; Randall L. Woltjer, MD PhD; Clinton B. Wright, MD MS; Steven G. Younkin, MD PhD; Chang-En Yu, PhD; Lei Yu, PhD;

GERAD: Alfredo Ramirez[2,3], Denise Harold[4], Michelle Lupton[5], Y. Patel[6], J.T. Hughes[6], Christopher Medway[7], Kristelle Brown[7], Jenny Lord[7], James Turton[7], Amy Gerrish[1], Nicola Denning[1] , Bernadette McGuinness[8], Stephen Todd[8], D. Craig[8], J. Johnston[8], Charlene Thomas[1] , Rhodri Thomas[1], Ana Frank-García[9,26], Ina Giegling[10], Harald Hampel[11], Wolfgang Maier[2,12], Per Hoffmann[3,13,14], Markus Nöthen[3,13], Martin Scherer[15] , Stefan Herms[3,13,14], Stefanie Heilmann[3,13], Susanne Moebus[16] , Tim Becker[12,17], Gianfranco Spalletta[18], Paola Bossù[18], Carlo Caltagirone[18], Maria Donata Orfei[18], Francesca Salani[18], Eleonora Sacchinelli[18], Onofre Combarros[19], Pascual Sánchez-Juan[19], Sara Ortega-Cubero[20,21], John Morris[22,23], Chris Corcoran[24], JoAnn Tschanz[24], Maria Norton[24], Ron Munger[24], María J. Bullido[21,25,26], Eliecer Coto[27], Victoria Alvarez[27], Simon Lovestone[28] , Matthias Riemenschneider[29], Sabrina Pichler[29], Manuel Mayhaus[29], Wei Gu[29], Carol Brayne[30], David C. Rubinsztein[31], Petroula Proitsi[6], Ammar Al-Chalabi[6], Christopher E. Shaw[6], Michael Gill[32], Brian Lawlor[32], Aoibhinn Lynch[32], Nick Fox[33], John Collinge[33], Magda Tsolaki[34], Alberto Lleó[35,36], Juan Fortea[35,36], Rafael Blesa[35,36], Jordi Clarimón[35,36], Rebecca Sussams[37], Alison Goate[22,23], Michael C. O'Donovan[1], Michael J. Owen[1], Simon Mead[33], Clive Holmes[37], John Powell[6], Kevin Morgan[7], Peter Passmore[8], Dan Rujescu[38], Pau Pastor[20,21,39], Carlos Cruchaga[22,23], John S.K. Kauwe[40], Peter A Holmans[1], Valentina Escott-Price[1]. **Affiliations of GERAD collaborators**: 1. Institute of Psychological Medicine and Clinical Neurosciences, MRC Centre for Neuropsychiatric Genetics & Genomics, Cardiff University, UK. 2. Department of Psychiatry and Psychotherapy, University of Bonn, 53127, Bonn, Germany. 3. Institute of Human Genetics, University of Bonn, 53127, Bonn, Germany. 4. Neuropsychiatric Genetics Group, Department of Psychiatry, Trinity Centre for Health Sciences, St James's Hospital, Dublin, Ireland. 5. QIMR Berghofer Medical Research Institute, Herston, Queensland, Australia. 6. Institute of Psychiatry, Psychology and Neuroscience, UK. 7. Institute of Genetics, Queen's Medical Centre, University of Nottingham, Nottingham, UK. 8. Ageing Group, Centre for Public Health, School of Medicine, Dentistry and Biomedical Sciences, Queen's University, Belfast, UK. 9. Department of Neurology, University Hospital La Paz. Universidad Autónoma de Madrid, Spain. 10. Dept. of Psychiatry, University of Halle, Germany. 11. Département de Neurologie, Hôpital Pitié Salpêtrière, Paris, France. 12. German Center for Neurodegenerative Diseases (DZNE), 53175, Bonn, Germany. 13. Department of Genomics, Life & Brain Center, University of Bonn, 53127, Bonn, Germany. 14. Division of Medical Genetics, University Hospital

and Department of Biomedicine, University of Basel, CH-4058, Basel, Switzerland. 15. Department of Primary Medical Care, University Medical Centre Hamburg-Eppendorf, 20246, Hamburg, Germany. 16. Institute for Medical Informatics, Biometry and Epidemiology, University Hospital of Essen, University Duisburg-Essen, Hufelandstr. 55, D-45147 Essen, Germany. 17. Institute for Medical Biometry, Informatics and Epidemiology, University of Bonn, Bonn, Germany. 18. Neuropsychiatry Laboratory, IRCCS Santa Lucia Foundation, Rome, Italy. 19. Neurology Service and CIBERNED, Marques de Valdecilla University Hospital (University of Cantabria and IDIVAL), Santander, Spain. 20. Neurogenetics Laboratory, Division of Neurosciences, Center for Applied Medical Research, University of Navarra School of Medicine, Pamplona, Spain. 21. CIBERNED, Centro de Investigación Biomédica en Red de Enfermedades Neurodegenerativas, Instituto de Salud Carlos III, Madrid, Spain. 22. Department of Psychiatry, Washington University School of Medicine, St. Louis, Missouri, USA. 23. Hope Center Program on Protein Aggregation and Neurodegeneration, Washington University School of Medicine, St. Louis, Missouri, USA. 24. Utah State University, Logan, Utah, USA. 25.Centro de Biologia Molecular Severo Ochoa (CSIC-UAM), Madrid, Spain. 26. IdiPAZ, Instituto de Investigación Sanitaria la Paz, Spain. 27. Molecular Genetics Dept, Hospital Universitario Central Asturias, 33011-Oviedo, Spain. 28. Department of Psychiatry, University of Oxford, Warneford Hospital, Oxford, UK. 29. Dept. Of Psychiatry and Psychotherapy, University Hospital, Saarland, Germany. 30. Institute of Public Health, University of Cambridge, Cambridge, UK. 31. Cambridge Institute for Medical Research, University of Cambridge, Cambridge, UK. 32. Mercer's Institute for Research on Aging, St. James Hospital and Trinity College, Dublin, Ireland. 33. MRC Prion Unit, Department of Neurodegenerative Disease, UCL Institute of Neurology, London, UK. 34. Department of Neurology, Aristotle University of Thessaloniki, Thessaloniki, Greece. 35. Memory Unit, Neurology Department and Sant Pau Biomedical Research Institute, Hospital de la Santa Creu i Sant Pau, Universitat Autònoma de Barcelona, Barcelona, Spain. 36. Center for Networking Biomedical Research in Neurodegenerative Diseases (CIBERNED), Madrid, Spain. 37. Division of Clinical Neurosciences, School of Medicine, University of Southampton, Southampton, UK. 38. Department of Psychiatry, Psychotherapy and Psychosomatics Martin-Luther-University Halle-Wittenberg, Julius-Kühn-Str. 706112 Halle Germany. 39. Memory Unit, Department of Neurology, Hospital Universitari Mutua de Terrassa, Terrassa, Barcelona, Spain. 40. Departments of Biology, Neuroscience, Brigham Young University, 4146 LSB Provo, UT 84602, USA.

Funding information

CHARGE: Infrastructure for the CHARGE Consortium is supported in part by the National Heart, Lung, and Blood Institute grant HL105756. Funding support for the CHARGE Consortium Exome Chip analyses is provided in part by the National Heart, Lung, and Blood Institute grant HL120393. Support for centralized calling of the exome chip was provided by Building on GWAS for NHLBI-diseases: the U.S. CHARGE consortium through the National Institutes of Health (NIH) American Recovery and Reinvestment Act of 2009 (ARRA) (5RC2HL102419). The CHARGE consortium is a founding component of the Alzheimer's Disease Sequencing Project, and receives sequencing and analysis support through the grants: U01-AG049506 to Eric Boerwinkle, U01-AG049505 to Sudha Seshadri, and U54-HG003273 to Richard Gibbs and Eric Boerwinkle.
AGES: This study has been funded by NIA contract N01-AG-12100 with contributions from NEI, NIDCD and NHLBI, the NIA Intramural Research Program, Hjartavernd (the Icelandic Heart Association), and the Althingi (the Icelandic Parliament).
Baylor College of Medicine: J.M.S. was supported by grants from the NIH/NIA (R01AG033193, R01AG050631, C06RR029965), the Alzheimer's Association, the American Federation for Aging Research, Huffington Foundation, Jan and Dan Duncan Neurological Research Institute at Texas Children's Hospital, and a Career Award for Medical Scientists from the Burroughs Wellcome Fund. The work was additionally supported by U54HD083092 from the Eunice Kennedy Shriver National Institute of Child Health & Human Development. S.Y. was supported by the Jan and Dan Duncan Neurological Research Institute at Texas Children's Hospital and the Alzheimer's Association. H.J.B. is a Howard Hughes Medical Institute Investigator and also received support from the Robert and Renee Belfer Family Foundation, the Huffington Foundation, and Target ALS. Jose Salazar is supported by NIH GMR2556929.
CHS: Cardiovascular Health Study: Cardiovascular Health Study: This CHS research was supported by NHLBI contracts HHSN268201200036C, HHSN268200800007C, N01HC55222, N01HC85079, N01HC85080, N01HC85081, N01HC85082, N01HC85083, N01HC85086; and NHLBI grants HL080295, HL087652, HL105756, HL103612, HL068986, and HL120393 with additional contribution from the National Institute of Neurological Disorders and Stroke (NINDS). Additional support was provided through AG023629, R01AG15928, and R01AG20098 from the National Institute on Aging (NIA). A full list of principal CHS investigators and institutions can be found at CHS-NHLBI.org/. The provision of genotyping data was supported in part by the National Center for Advancing Translational Sciences, CTSI grant UL1TR000124, and the National Institute of Diabetes and Digestive and Kidney Disease Diabetes Research Center (DRC) grant DK063491 to the Southern California Diabetes Endocrinology Research Center. The content is solely the

responsibility of the authors and does not necessarily represent the official views of the National Institutes of Health.
FHS: Framingham Heart Study: This work was supported by the National Heart, Lung and Blood Institute's Framingham Heart Study (Contract No. N01-HC-25195 and No. HHSN268201500001I). This study was also supported by grants from the National Institute on Aging: AG033193, U01-AG049505 and AG008122 (Seshadri). Drs. Seshadri and DeStefano were also supported by additional grants from the National Institute on Aging (R01AG049607), the National Institute of Neurological Disorders and Stroke (R01-NS017950).
RS: The Rotterdam Study is funded by Erasmus Medical Center and Erasmus University, Rotterdam, Netherlands Organization for the Health Research and Development (ZonMw), the Research Institute for Diseases in the Elderly (RIDE), the Ministry of Education, Culture and Science, the Ministry for Health, Welfare and Sports, the European Commission (DG XII), and the Municipality of Rotterdam. The authors are grateful to the study participants, the staff from the Rotterdam Study and the participating general practitioners and pharmacists. The generation and management of the Illumina exome chip v1.0 array data for the Rotterdam Study (RS-I) was executed by the Human Genotyping Facility of the Genetic Laboratory of the Department of Internal Medicine, Erasmus MC, Rotterdam, The Netherlands. The Exome chip array data set was funded by the Genetic Laboratory of the Department of Internal Medicine, Erasmus MC, from the Netherlands Genomics Initiative (NGI)/Netherlands Organisation for Scientific Research (NWO)-sponsored Netherlands Consortium for Healthy Aging (NCHA; project nr. 050-060-810); the Netherlands Organization for Scientific Research (NWO; project number 184021007) and by the Rainbow Project (RP10; Netherlands Exome Chip Project) of the Biobanking and Biomolecular Research Infrastructure Netherlands (BBMRI-NL; www.bbmri.nl (http://www.bbmri.nl)). We thank Ms. Mila Jhamai, Ms. Sarah Higgins, and Mr. Marijn Verkerk for their help in creating the exome chip database, and Carolina Medina-Gomez, MSc, Lennard Karsten, MSc, and Linda Broer, PhD, for QC and variant calling. The generation and management of the exome sequencing data for the Rotterdam Study was executed by the Human Genotyping Facility of the Genetic Laboratory of the Department of Internal Medicine, Erasmus MC, the Netherlands. The Exome Sequencing data set was funded by the Netherlands Genomics Initiative (NGI)/Netherlands Organisation for Scientific Research (NWO) sponsored Netherlands Consortium for Healthy Aging (NCHA; project nr. 050-060-810), by the Genetic Laboratory of the Department of Internal Medicine, Erasmus MC, and by the and by a Complementation Project of the Biobanking and Biomolecular Research Infrastructure Netherlands (BBMRI-NL; www.bbmri.nl ; project number CP2010-41). We thank Mr. Pascal Arp, Ms. Mila Jhamai, Jeroen van Rooij, MSc, Mr. Marijn Verkerk, and Robert Kraaij, PhD, for their help in creating the RS-Exome Sequencing database.
ADGC: The Alzheimer's Disease Genetics Consortium is funded by the U.S. National Institutes of Health, National Institute on Aging (NIH-NIA) grants U01-

AG032984 and RC2-AG036528 and a grant from a private foundation wishing to remain anonymous. The NIH-NIA also provides financial support to NIAGADS (U24-AG041689), NACC (U01-AG016976), NCRAD (U24-AG021886), and the Alzheimer's Disease Centers: Arizona (P30 AG019610), Boston University (P30 AG013846, R01 HG02213, K24 AG027841, U01 AG10483, R01 CA129769, R01 MH080295), Columbia University (P50 AG008702), Duke University (P30 AG028377), Emory University (AG025688), Indiana University (P30 AG10133), Johns Hopkins University (P50 AG005146), Massachusetts General Hospital (P50 AG005134), Mayo Clinic (P50 AG016574), Mount Sinai School of Medicine (P50 AG005138), New York University (P30 AG08051, U01 AG16976, MO1 RR00096, and UL1 RR029893), Northwestern University (P30 AG013854), Oregon Health & Science University (P30 AG008017), Rush University (P30 AG010161), University of Alabama at Birmingham (P50 AG016582, UL1 RR02777 through the UAB Center for Clinical and Translational Science), University of California, Davis (P30 AG010129), University of California, Irvine (P50 AG016573, P50 AG016574, P50 AG016575, P50 AG016576, P50 AG016577), University of California, Los Angeles (P50 AG016570), University of California, San Diego (P50 AG005131), University of California, San Francisco (P50 AG023501, P01 AG019724), University of Kentucky (P30 AG028383), University of Michigan (P50 AG008671), University of Pennsylvania (P30 AG010124), University of Pittsburgh (P50 AG005133), University of Southern California (P50 AG005142), University of Texas Southwestern (P30 AG012300), University of Washington (P50 AG005136 and R01 AG007584), and the Washington University (P50 AG005681 and P01 AG03991). The work completed by Boston University is also supported by the Alzheimer’s Association (IIRG-08-89720) and VA New England Geriatric Research Education and Clinical Center. The work by Miami University is also supported by R01AG027944 and a grant from the Alzheimer’s Association. We thank Drs. Creighton Phelps, Ph.D., Stephen Snyder, Ph.D., and Marilyn Miller Ph.D. from the National Institute on Aging for helping in acquiring samples and data and who are ex-officio members of the ADGC. Duke University would also like to acknowledge John Ervin from the Brain Bank and Kathleen Hayden in the Clinical Core for their respective efforts in the DNA/data pulls required. This project was also made possible by the many contributions of individual study datasets, supported in part by NIH. These include ACT and eMERGE (NIH U01 AG06781, U01 HG004610 and U01 HG006375 from the National Human Genome Research Institute), NIA LOAD (NIH U24 AG026395 and R01 AG041797), Columbia University WHICAP (NIH R01 AG037212),and MIRAGE (R01 AG009029). The UM/VU/MSSM work was supported by grants from the NIA-NIH (AG010491, AG002219, AG005138, AG027944, AG021547, AG019757 and R01-AG-027944) and from the Alzheimer’s Association (IIRG-05-14147). A subset of these participants was ascertained while Dr. Margaret A. Pericak-Vance was a faculty member at Duke University. Clinical data and genotyping efforts for ROS and MAP supported by the following grants from NIA: P30AG10161, R01AG15819, R01AG17917,

R01AG30146, the Illinois Department of Public Health, and the Translational Genomics Research Institute. Data and samples from the National Institute on Aging – Late Onset Alzheimer's Disease (NIA-LOAD) Family Study, which receives government support under a cooperative agreement grant (U24AG026390), were used in this study. Data from the Cache County Study on Memory in Aging was supported by grants to Brigham Young University by the NIH-NIA (R01 AG042611), the Alzheimer's Association (MNIRG-11-205368), the Brigham Young University Gerontology Program and the Charleston Conference on Alzheimer's Disease and to Utah State University (NIH: R01 AG11380, R01 AG21136, R01AG3272; the Utah Science, Technology, and Research Initiative, and the Utah State University Agricultural Experiment Station). The following investigators and Alzheimer's Disease Centers participated in the NIA-LOAD Family Study: Boston University Neil Kowall; Columbia University Jennifer Williamson, Vincent Santana; Duke University Donald Schmechel, Perry Gaskell, Kathleen Welsh-Bohmer; Indiana University, Bernardino Ghetti, Martin R.Farlow, Kelly Horner; Massachusetts General Hospital John H. Growdon, Deborah Blacker, Rudolph E. Tanzi, Bradley T. Hyman; Mayo Clinic-Rochester Bradley Boeve, Karen Kuntz, Lindsay Norgaard, Nathan Larson; Mayo Clinic-Jacksonville Dana Kistler, Fracine Parfitt, Jenny Haddwow; Mount Sinai School of Medicine Jeremy Silverman, Michal Schnaider Beeri, Mary Sano, Joy Wang, Rachel Lally; Northwestern University Nancy Johnson, Marcel Mesulum, Sandra Weintraub, Eileen Bigio; Oregon Health and Science University Jeffery Kaye, Patricia Kramer, Jessica Payne-Murphy; Rush University David Bennett, Holli Jacobs, Jeen-Soo Chang, Danielle Arends; University of Alabama at Birmingham Lindy Harrell; University of California, Los Angeles George Bartzokis, Jeffery Cummings, Po H Lu, Usha Toland; University of Kentucky William Markesbery, Charles Smith, Alise Brickhouse; University of Pennsylvania John Trojanowski, Vivianna Van Deerlin, Elisabeth McCarty Wood; University of Pittsburgh Steven DeKosky, Robert Sweet, Elise Weamer; University of Southern California I Helena Chui, Arousiak Varpetian; University of Texas Southwestern Ramon Diaz-Arrastia, Roger Rosenberg, Barbara Davis; University of Washington Thomas Bird, Malia Rumbaugh, Gerard D. Schellenberg, Murray Raskind; Washington University at St Louis Alison Goate, John Morris, Joanne Norton, Denise Levitch, Betsy Grant, Mary Coats. Samples from the National Cell Repository for Alzheimer's Disease (NCRAD), which receives government support under a cooperative agreement grant (U24 AG21886) awarded by the National Institute on Aging (NIA), were used in this study. GDS and L-SW had full access to all the data in the study and take responsibility for the integrity of the data and the accuracy of the data analysis. We thank Annette Lee and Peter K. Gregersen at the Feinstein Institute for Medical Research for genotyping services. We thank contributors, including the Alzheimer's Disease Centers who collected samples used in this study, as well as patients and their families, whose help and participation made this work possible.

GERAD: We thank all the individuals who participated in this study. Cardiff University was supported by the Alzheimer's Society (AS) and the Medical Research Council (MRC) (Rebecca Sims is an AS Research Fellow). Cambridge University acknowledges support from the MRC. Patient recruitment for the MRC Prion Unit/UCL Department of Neurodegenerative Disease collection was supported by the UCLH/UCL Biomedical Research Centre and Queen Square Dementia Biomedical Research Unit. The University of Southampton acknowledges support from the AS. King's College London were supported by the National Institutes for Health Research Biomedical Research Centre for Mental Health at the South London and the Maudsley National Health Service Foundation Trust, the Institute of Psychiatry and the MRC. Alzheimer's Research UK (ARUK) and the Big Lottery Fund provided support to Nottingham University. Ulster Garden Villages, AS, ARUK, American Federation for Aging Research and NI R&D Office provided support for Queen's University, Belfast. The Centro de Biología Molecular Severo Ochoa (CSIC-UAM), CIBERNED, Instituto de Investigación Sanitaria la Paz, University Hospital La Paz and the Universidad Autónoma de Madrid were supported by grants from the Ministerio de Educación y Ciencia and the Ministerio de Sanidad y Consumo (Instituto de Salud Carlos III), and an institutional grant of the FundaciónRamón Areces to the CBMSO. Thanks to I. Sastre and Dr. A Martínez-García for DNA preparation, and Drs. P. Gil and P. Coria for their recruitment efforts. Universitari Mutua de Terrassa, Terrassa, Barcelona, University of Navarra School of Medicine, Pamplona, Spain and CIBERNED, Centro de Investigación Biomédica en Red de Enfermedades Neurodegenerativas, Instituto de Salud Carlos III, Madrid, Spain were partially supported by the FIMA (Foundation for Applied Medical Research) and acknowledge Maria A. Pastor (Department of Neurology, University of Navarra Medical School and Neuroimaging Laboratory, Center for Applied Medical Research, Pamplona, Spain), Manuel Seijo-Martínez (Department of Neurology, Hospital do Salnés, Pontevedra, Spain), Ramon Rene, Jordi Gascon and Jaume Campdelacreu (Department of Neurology, Hospital de Bellvitge, Barcelona, Spain) for providing DNA samples. Hospital de la Sant Pau, Universitat Autònoma de Spain acknowledges support from the Spanish Ministry of Economy and Competitiveness [grant number PI12/01311]. The Santa Lucia Foundation, Italy, acknowledges a grant from the Italian Ministry of Health (RC 10.11.12.13/A). The Bonn samples are part of the German Dementia Competence Network (DCN) and the German Research Network on Degenerative Dementia (KNDD), which are funded by the German Federal Ministry of Education and Research (grants KND: 01GI0102, 01GI0420, 01GI0422, 01GI0423, 01GI0429, 01GI0431, 01GI0433, 01GI0434; grants KNDD: 01GI1007A, 01GI0710, 01GI0711, 01GI0712, 01GI0713, 01GI0714, 01GI0715, 01GI0716, 01ET1006B). Markus M. Nöthen is member of the German Research Foundation (DFG) cluster of excellence ImmunoSensation. Funding for Saarland University was provided by the German Federal Ministry of Education and research (BMBF), Grant No. 01GS08125 to Matthias Riemenschneider. The

University of Washington was supported by grants from the National Institutes of Health (R01-NS085419) and R01-AG044546), the Alzheimer Association (NIRG-11-200110) and the American Federation for Aging Research (Carlos Cruchaga was a recipient of a New Investigator Award in Alzheimer's disease). Brigham Young University was supported by the Alzheimer's Association (MNIRG-11-205368), the BYU Gerontology Program and the National Institutes of Health (R01-AG11380, R01- AG021136, P30-NS069329-01, R01-AG042611).
Generation Scotland: Generation Scotland (IRAS id:178140) received core funding from the Chief Scientist Office of the Scottish Government Health Directorate CZD/16/6 and the Scottish Funding Council HR03006. Genotyping of the GS:SFHS samples was carried out by the Genetics Core Laboratory at the Wellcome Trust Clinical Research Facility, Edinburgh, Scotland and was funded by the UK's Medical Research Council. Ethics approval for the study was given by the NHS Tayside committee on research ethics (reference 05/S1401/89). We are grateful to all the families who took part, the general practitioners and the Scottish School of Primary Care for their help in recruiting them, and the whole Generation Scotland team, which includes interviewers, computer and laboratory technicians, clerical workers, research scientists, volunteers, managers, receptionists, healthcare assistants and nurses.
GLACIER/DIABNORD: We thank the study participants who dedicated their time and samples to these studies. We thank J. Hutiainen and Å. Ågren (Northern Sweden Biobank) for data organization and K. Enquist and T. Johansson (Västerbottens County Council) for technical assistance with DNA extraction. We also thank M. Sterner, G. Gramsperger and P. Storm for their expert technical assistance with genotyping and genotype data preparation. The current study was funded by Novo Nordisk, the Swedish Research Council, Påhlssons Foundation, the Swedish Heart Lung Foundation, and the Skåne Regional Health Authority (all to P. W. Franks).
FIA3: We are indebted to the study participants who dedicated their time and samples to these studies. We thank Å. Ågren (Northern Sweden Biobank) for data organization and K. Enquist and T. Johansson (Västerbottens County Council) for technical assistance with DNA extraction. P. Deluokas' work forms part of the research themes contributing to the translational research portfolio of Barts Cardiovascular Biomedical Research Unit which is supported and funded by the National Institute for Health Research.
FINRISK: The FINRISK surveys were mainly funded by budgetary funds from the National Institute for Health and Welfare. Additional funding has been obtained from the Academy of Finland (grant #139635 to Veikko Salomaa) and from the Finnish Foundation for Cardiovascular Research. Samuli Ripatti was financially supported by the Academy of Finland [251217 and 255847], EU FP7 projects ENGAGE (201413), BioSHaRE (261433), the Finnish Foundation for Cardiovascular Research, Biocentrum Helsinki, and the Sigrid Juselius Foundation.

ESP: The authors would like to thank the NHLBI GO Exome Sequencing Project and its ongoing studies which produced and provided exome variant calls for comparison: the Lung GO Sequencing Project (HL-102923), the WHI Sequencing Project (HL-102924), the Broad GO Sequencing Project (HL-102925), the Seattle GO Sequencing Project (HL-102926) and the Heart GO Sequencing Project (HL-103010).
GTEx: The Genotype-Tissue Expression (GTEx) Project was supported by the Common Fund (http://commonfund.nih.gov/GTEx/index) of the Office of the Director of the National Institutes of Health. Additional funds were provided by the NCI, NHGRI, NHLBI, NIDA, NIMH, and NINDS. Donors were enrolled at Biospecimen Source Sites funded by NCI\SAIC-Frederick, Inc. (SAIC-F) subcontracts to the National Disease Research Interchange (10XS170), Roswell Park Cancer Institute (10XS171), and Science Care, Inc. (X10S172). The Laboratory, Data Analysis, and Coordinating Center (LDACC) was funded through a contract (HHSN268201000029C) to The Broad Institute, Inc. Biorepository operations were funded through an SAIC-F subcontract to Van Andel Institute (10ST1035). Additional data repository and project management were provided by SAIC-F (HHSN261200800001E). The Brain Bank was supported by a supplements to University of Miami grants DA006227 & DA033684 and to contract N01MH000028. Statistical Methods development grants were made to the University of Geneva (MH090941 & MH101814), the University of Chicago (MH090951, MH090937, MH101820, MH101825), the University of North Carolina - Chapel Hill (MH090936 & MH101819), Harvard University (MH090948), Stanford University (MH101782), Washington University St Louis (MH101810), and the University of Pennsylvania (MH101822). The data used for the analyses described in this manuscript were obtained from the UCSC Xena browser (https://xena.ucsc.edu/) using the TOIL workflow (https://genome-cancer.soe.ucsc.edu/proj/site/xena/datapages/?host=https://toil.xenahubs.net.) on 05/25/2016.
ExAC: The authors would like to thank the Exome Aggregation Consortium and the groups that provided exome variant data for comparison. A full list of contributing groups can be found at http://exac.broadinstitute.org/about.

Supporting references

1. Ma C, Blackwell T, Boehnke M, Scott LJ. Recommended joint and meta-analysis strategies for case-control association testing of single low-count variants. Genet Epidemiol. 2013;37: 539–50. doi:10.1002/gepi.21742

2. Zhong H, Prentice RL. Correcting “winner’s curse” in odds ratios from genomewide association findings for major complex human diseases. Genet Epidemiol. 2010;34: 78–91. doi:10.1002/gepi.20437

3. Stephens M, Scheet P. Accounting for decay of linkage disequilibrium in haplotype inference and missing-data imputation. Am J Hum Genet. 2005;76: 449–62. doi:10.1086/428594

4. Stephens M, Donnelly P. A comparison of bayesian methods for haplotype reconstruction from population genotype data. Am J Hum Genet. 2003;73: 1162–9. doi:10.1086/379378

5. Stephens M, Smith NJ, Donnelly P. A new statistical method for haplotype reconstruction from population data. Am J Hum Genet. 2001;68: 978–89. doi:10.1086/319501

6. Mathieson I, McVean G. Differential confounding of rare and common variants in spatially structured populations. Nat Genet. 2012;44: 243–6. doi:10.1038/ng.1074

7. Shannon MP. Characterization of the female-sterile mutant Almondex of Drosophila melanogaster. Genetica. 1972;43: 244–256. doi:10.1007/BF00123632

8. Parks AL, Cook KR, Belvin M, Dompe NA, Fawcett R, Huppert K, et al. Systematic generation of high-resolution deletion coverage of the Drosophila melanogaster genome. Nat Genet. 2004;36: 288–92. doi:10.1038/ng1312

9. Venken KJT, He Y, Hoskins RA, Bellen HJ. P[acman]: a BAC transgenic platform for targeted insertion of large DNA fragments in D. melanogaster. Science. 2006;314: 1747–51. doi:10.1126/science.1134426

10. Venken KJT, Carlson JW, Schulze KL, Pan H, He Y, Spokony R, et al. Versatile P[acman] BAC libraries for transgenesis studies in Drosophila melanogaster. Nat Methods. 2009;6: 431–4. doi:10.1038/nmeth.1331

11. Bischof J, Maeda RK, Hediger M, Karch F, Basler K. An optimized transgenesis system for Drosophila using germ-line-specific phiC31 integrases. Proc Natl Acad Sci U S A. 2007;104: 3312–7. doi:10.1073/pnas.0611511104

12. Engler C, Kandzia R, Marillonnet S. A one pot, one step, precision cloning

method with high throughput capability. PLoS One. 2008;3: e3647. doi:10.1371/journal.pone.0003647

13. Engler C, Gruetzner R, Kandzia R, Marillonnet S. Golden gate shuffling: a one-pot DNA shuffling method based on type IIs restriction enzymes. PLoS One. 2009;4: e5553. doi:10.1371/journal.pone.0005553

14. Snow PM, Patel NH, Harrelson AL, Goodman CS. Neural-specific carbohydrate moiety shared by many surface glycoproteins in Drosophila and grasshopper embryos. J Neurosci. 1987;7: 4137–44. Available: http://www.ncbi.nlm.nih.gov/pubmed/3320283

15. Robinow S, White K. Characterization and spatial distribution of the ELAV protein during Drosophila melanogaster development. J Neurobiol. 1991;22: 443–61. doi:10.1002/neu.480220503

16. Harris TB, Launer LJ, Eiriksdottir G, Kjartansson O, Jonsson P V, Sigurdsson G, et al. Age, Gene/Environment Susceptibility-Reykjavik Study: multidisciplinary applied phenomics. Am J Epidemiol. 2007;165: 1076–87. doi:10.1093/aje/kwk115

17. American Psychiatric Association. DSM-IV: Diagnostic and Statistical Manual of Mental Disorders [Hardcover]. American Psychiatric Association; 1994.

18. Morris JN, Hawes C, Fries BE, Phillips CD, Mor V, Katz S, et al. Designing the national resident assessment instrument for nursing homes. Gerontologist. 1990;30: 293–307. Available: http://www.ncbi.nlm.nih.gov/pubmed/2354790

19. Morris JN, Fries BE, Steel K, Ikegami N, Bernabei R, Carpenter GI, et al. Comprehensive clinical assessment in community setting: applicability of the MDS-HC. J Am Geriatr Soc. 1997;45: 1017–24. Available: http://www.ncbi.nlm.nih.gov/pubmed/9256857

20. Sgadari A, Morris JN, Fries BE, Ljunggren G, Jónsson P V, DuPaquier JN, et al. Efforts to establish the reliability of the Resident Assessment Instrument. Age Ageing. 1997;26 Suppl 2: 27–30. Available: http://www.ncbi.nlm.nih.gov/pubmed/9464551

21. Jonsson P V. RAI experience in Iceland, at http://www.milbank.org/uploads/documents/interRAI/030222interRAI.html#iceland [Internet]. 2003. Available: http://www.milbank.org/uploads/documents/interRAI/030222interRAI.html#iceland

22. McKhann G, Drachman D, Folstein M, Katzman R, Price D, Stadlan EM. Clinical diagnosis of Alzheimer’s disease: report of the NINCDS-ADRDA Work Group under the auspices of Department of Health and Human Services Task Force on Alzheimer's Disease. Neurology. 1984;34: 939–44.

Available: http://www.ncbi.nlm.nih.gov/pubmed/6610841

23. Fried LP, Borhani NO, Enright P, Furberg CD, Gardin JM, Kronmal RA, et al. The Cardiovascular Health Study: design and rationale. Ann Epidemiol. 1991;1: 263–76. Available: http://www.ncbi.nlm.nih.gov/pubmed/1669507

24. Purcell S, Neale B, Todd-Brown K, Thomas L, Ferreira MAR, Bender D, et al. PLINK: a tool set for whole-genome association and population-based linkage analyses. Am J Hum Genet. 2007;81: 559–75. doi:10.1086/519795

25. Fitzpatrick AL, Kuller LH, Ives DG, Lopez OL, Jagust W, Breitner JCS, et al. Incidence and prevalence of dementia in the Cardiovascular Health Study. J Am Geriatr Soc. 2004;52: 195–204. Available: http://www.ncbi.nlm.nih.gov/pubmed/14728627

26. Lopez OL, Kuller LH, Fitzpatrick A, Ives D, Becker JT, Beauchamp N. Evaluation of dementia in the cardiovascular health cognition study. Neuroepidemiology. 2003;22: 1–12. doi:67110

27. Kannel WB. Factors of Risk in the Development of Coronary Heart Disease—Six-Year Follow-up Experience. Ann Intern Med. American College of Physicians; 1961;55: 33. doi:10.7326/0003-4819-55-1-33

28. Feinleib M, Kannel WB, Garrison RJ, McNamara PM, Castelli WP. The Framingham Offspring Study. Design and preliminary data. Prev Med (Baltim). 1975;4: 518–25. Available: http://www.ncbi.nlm.nih.gov/pubmed/1208363

29. Splansky GL, Corey D, Yang Q, Atwood LD, Cupples LA, Benjamin EJ, et al. The Third Generation Cohort of the National Heart, Lung, and Blood Institute's Framingham Heart Study: design, recruitment, and initial examination. Am J Epidemiol. 2007;165: 1328–35. doi:10.1093/aje/kwm021

30. Folstein MF, Folstein SE, McHugh PR. "Mini-mental state". A practical method for grading the cognitive state of patients for the clinician. J Psychiatr Res. 1975;12: 189–98. Available: http://www.ncbi.nlm.nih.gov/pubmed/1202204

31. Hofman A, Brusselle GGO, Darwish Murad S, van Duijn CM, Franco OH, Goedegebure A, et al. The Rotterdam Study: 2016 objectives and design update. Eur J Epidemiol. Springer; 2015;30: 661–708. doi:10.1007/s10654-015-0082-x

32. Hooijer C, van Tilburg W. [The Geriatric Mental Status Schedule, the GMS: psychiatric tool in psychogeriatrics]. Tijdschr Gerontol Geriatr. 1988;19: 103–11. Available: http://www.ncbi.nlm.nih.gov/pubmed/3394165

33. Roth M, Tym E, Mountjoy CQ, Huppert FA, Hendrie H, Verma S, et al. CAMDEX. A standardised instrument for the diagnosis of mental disorder

in the elderly with special reference to the early detection of dementia. Br J Psychiatry. 1986;149: 698–709. Available: http://www.ncbi.nlm.nih.gov/pubmed/3790869

34. American Psychiatric Association. Diagnostic and Statistical Manual of Mental Disorders: DSM-III-R: American Psychiatric Association: 9780890420188: Amazon.com: Books. American Psychiatric Association; 1987.

35. Grove ML, Yu B, Cochran BJ, Haritunians T, Bis JC, Taylor KD, et al. Best practices and joint calling of the HumanExome BeadChip: the CHARGE Consortium. PLoS One. 2013;8: e68095. doi:10.1371/journal.pone.0068095

36. Liu X, Jian X, Boerwinkle E. dbNSFP v2.0: a database of human non-synonymous SNVs and their functional predictions and annotations. Hum Mutat. 2013;34: E2393–402. doi:10.1002/humu.22376

37. Li H, Durbin R. Fast and accurate short read alignment with Burrows-Wheeler transform. Bioinformatics. 2009;25: 1754–60. doi:10.1093/bioinformatics/btp324

38. Li H, Handsaker B, Wysoker A, Fennell T, Ruan J, Homer N, et al. The Sequence Alignment/Map format and SAMtools. Bioinformatics. 2009;25: 2078–9. doi:10.1093/bioinformatics/btp352

39. McKenna A, Hanna M, Banks E, Sivachenko A, Cibulskis K, Kernytsky A, et al. The Genome Analysis Toolkit: a MapReduce framework for analyzing next-generation DNA sequencing data. Genome Res. 2010;20: 1297–303. doi:10.1101/gr.107524.110

40. Peloso GM, Auer PL, Bis JC, Voorman A, Morrison AC, Stitziel NO, et al. Association of low-frequency and rare coding-sequence variants with blood lipids and coronary heart disease in 56,000 whites and blacks. Am J Hum Genet. 2014;94: 223–32. doi:10.1016/j.ajhg.2014.01.009

41. Jun G, Naj AC, Beecham GW, Wang L-S, Buros J, Gallins PJ, et al. Meta-analysis confirms CR1, CLU, and PICALM as alzheimer disease risk loci and reveals interactions with APOE genotypes. Arch Neurol. NIH Public Access; 2010;67: 1473–84. doi:10.1001/archneurol.2010.201

42. Naj AC, Jun G, Beecham GW, Wang L-S, Vardarajan BN, Buros J, et al. Common variants at MS4A4/MS4A6E, CD2AP, CD33 and EPHA1 are associated with late-onset Alzheimer's disease. Nat Genet. 2011;43: 436–41. doi:10.1038/ng.801

43. Kukull WA, Schellenberg GD, Bowen JD, McCormick WC, Yu C-E, Teri L, et al. Apolipoprotein E in Alzheimer's disease risk and case detection: A case-control study. J Clin Epidemiol. 1996;49: 1143–1148. doi:10.1016/0895-4356(96)00195-3

44. Tang M-X. The APOE-ϵ4 Allele and the Risk of Alzheimer Disease Among African Americans, Whites, and Hispanics. JAMA. American Medical Association; 1998;279: 751. doi:10.1001/jama.279.10.751

45. Blacker D, Haines JL, Rodes L, Terwedow H, Go RCP, Harrell LE, et al. ApoE-4 and Age at Onset of Alzheimer's Disease: The NIMH Genetics Initiative. Neurology. 1997;48: 139–147. doi:10.1212/WNL.48.1.139

46. Naj AC, Beecham GW, Martin ER, Gallins PJ, Powell EH, Konidari I, et al. Dementia revealed: novel chromosome 6 locus for late-onset Alzheimer disease provides genetic evidence for folate-pathway abnormalities. PLoS Genet. Public Library of Science; 2010;6: e1001130. doi:10.1371/journal.pgen.1001130

47. Beecham GW, Martin ER, Li Y-J, Slifer MA, Gilbert JR, Haines JL, et al. Genome-wide association study implicates a chromosome 12 risk locus for late-onset Alzheimer disease. Am J Hum Genet. 2009;84: 35–43. doi:10.1016/j.ajhg.2008.12.008

48. Breitner JCS, Wyse BW, Anthony JC, Welsh-Bohmer KA, Steffens DC, Norton MC, et al. APOE- 4 count predicts age when prevalence of AD increases, then declines: The Cache County Study. Neurology. Lippincott Williams & Wilkins; 1999;53: 321–321. doi:10.1212/WNL.53.2.321

49. Zetterberg M, Landgren S, Andersson ME, Palmér MS, Gustafson DR, Skoog I, et al. Association of complement factor H Y402H gene polymorphism with Alzheimer's disease. Am J Med Genet Part B Neuropsychiatr Genet. 2008;147B: 720–726. doi:10.1002/ajmg.b.30668

50. Goldstein JI, Crenshaw A, Carey J, Grant GB, Maguire J, Fromer M, et al. zCall: a rare variant caller for array-based genotyping: genetics and population analysis. Bioinformatics. 2012;28: 2543–5. doi:10.1093/bioinformatics/bts479

51. Smith BH, Campbell H, Blackwood D, Connell J, Connor M, Deary IJ, et al. Generation Scotland: the Scottish Family Health Study; a new resource for researching genes and heritability. BMC Med Genet. 2006;7: 74. doi:10.1186/1471-2350-7-74

52. Hallmans G, Agren A, Johansson G, Johansson A, Stegmayr B, Jansson J-H, et al. Cardiovascular disease and diabetes in the Northern Sweden Health and Disease Study Cohort - evaluation of risk factors and their interactions. Scand J Public Health Suppl. 2003;61: 18–24. doi:10.1080/14034950310001432

53. Vartiainen E, Laatikainen T, Peltonen M, Juolevi A, Männistö S, Sundvall J, et al. Thirty-five-year trends in cardiovascular risk factors in Finland. Int J Epidemiol. 2010;39: 504–18. doi:10.1093/ije/dyp330
