# Supplementary figures and images for: Rare Functional Variant in TM2D3 is Associated with Late-Onset Alzheimer's Disease
Source: PLoS Genet. 2016 Oct 20;12(10):e1006327. doi: 10.1371/journal.pgen.1006327 (PMC5072721; doi:10.1371/journal.pgen.1006327)

SNPs in all genes

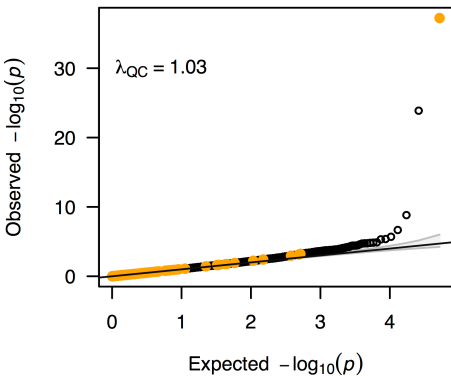

SNPs in all genes except APOE

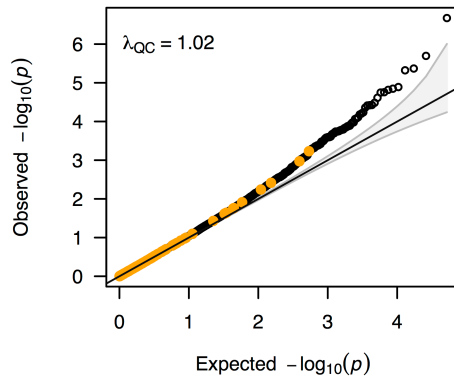

SKAT for all genes

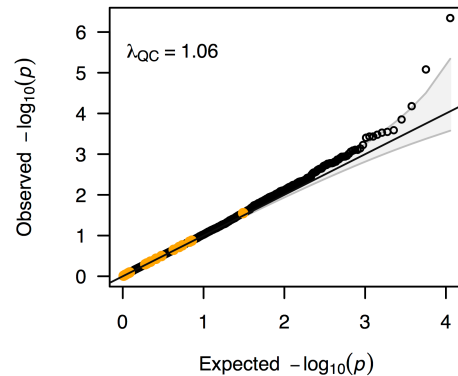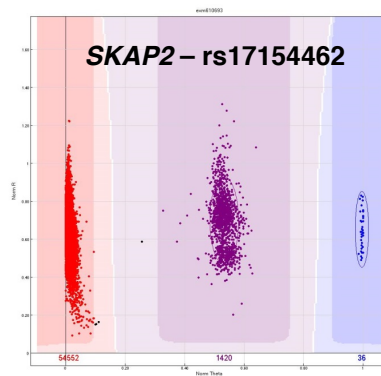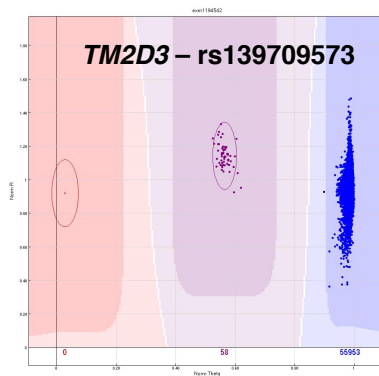

Supplement: S1 Fig — Top row Quantile-quantile plots for the exome-wide discovery meta-analysis. Known genes are in orange. The genomic control coefficient (λGC) is reported. Middle panel: Variants near APOE excluded Bottom row Cluster plots for SNPs in SKAP2 and TM2D3 demonstrate appropriate calling of the rare variant genotypes. (PDF) [file pgen.1006327.s002.pdf]

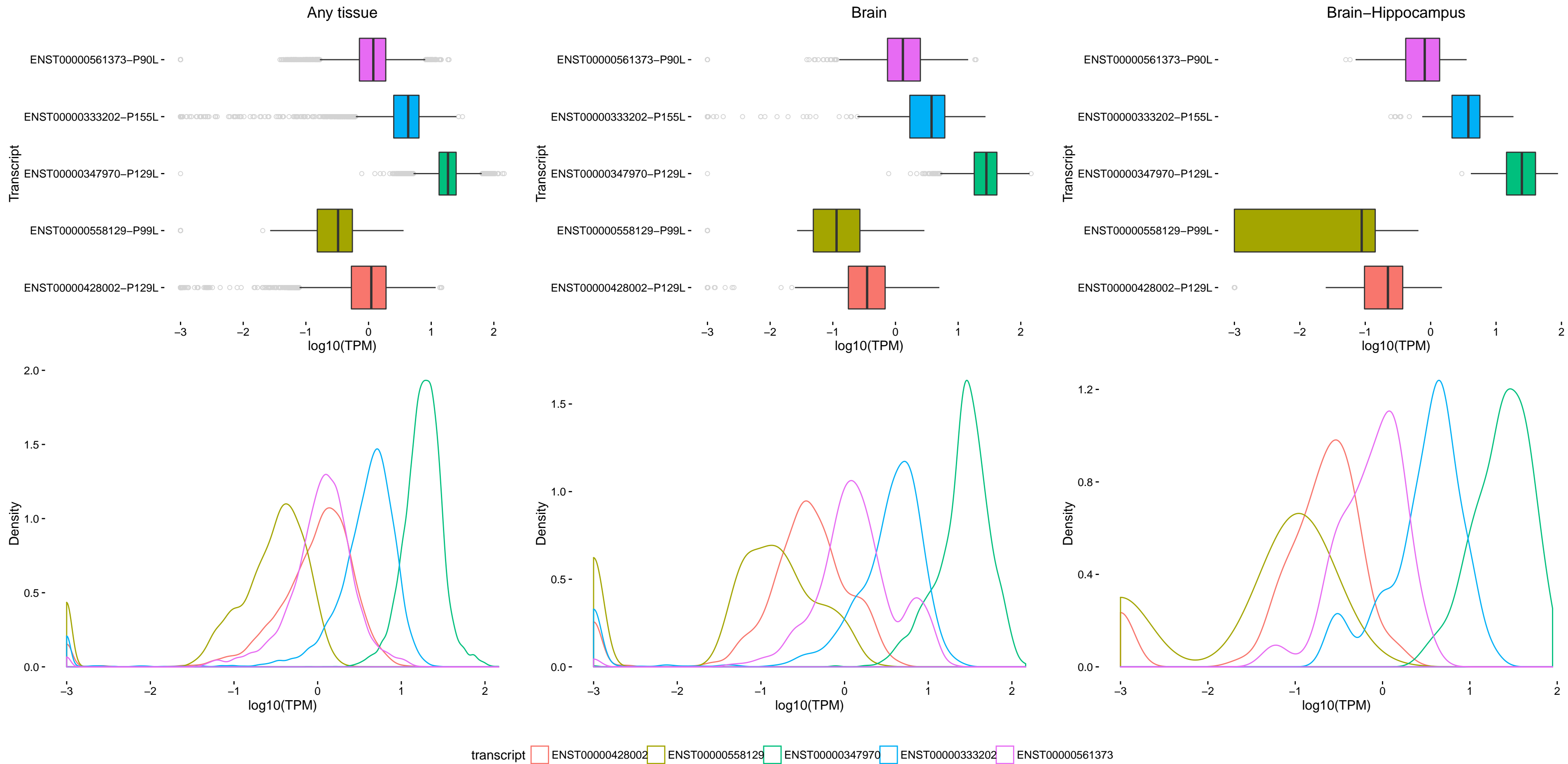

Supplement: S4 Fig — Expression of protein-coding transcripts in all tissues (excluding blood), all brain tissues, and hippocampus only. TPM (“transcripts per million) estimated using kallisto [59] on the GTEx data [37] (retrieved 26 May 2016 from UCSC Xena browser). Transcript ENST00000559107 was not included in the plots because it is very lowly expressed and visualization was better without it. (PDF) [file pgen.1006327.s005.pdf]

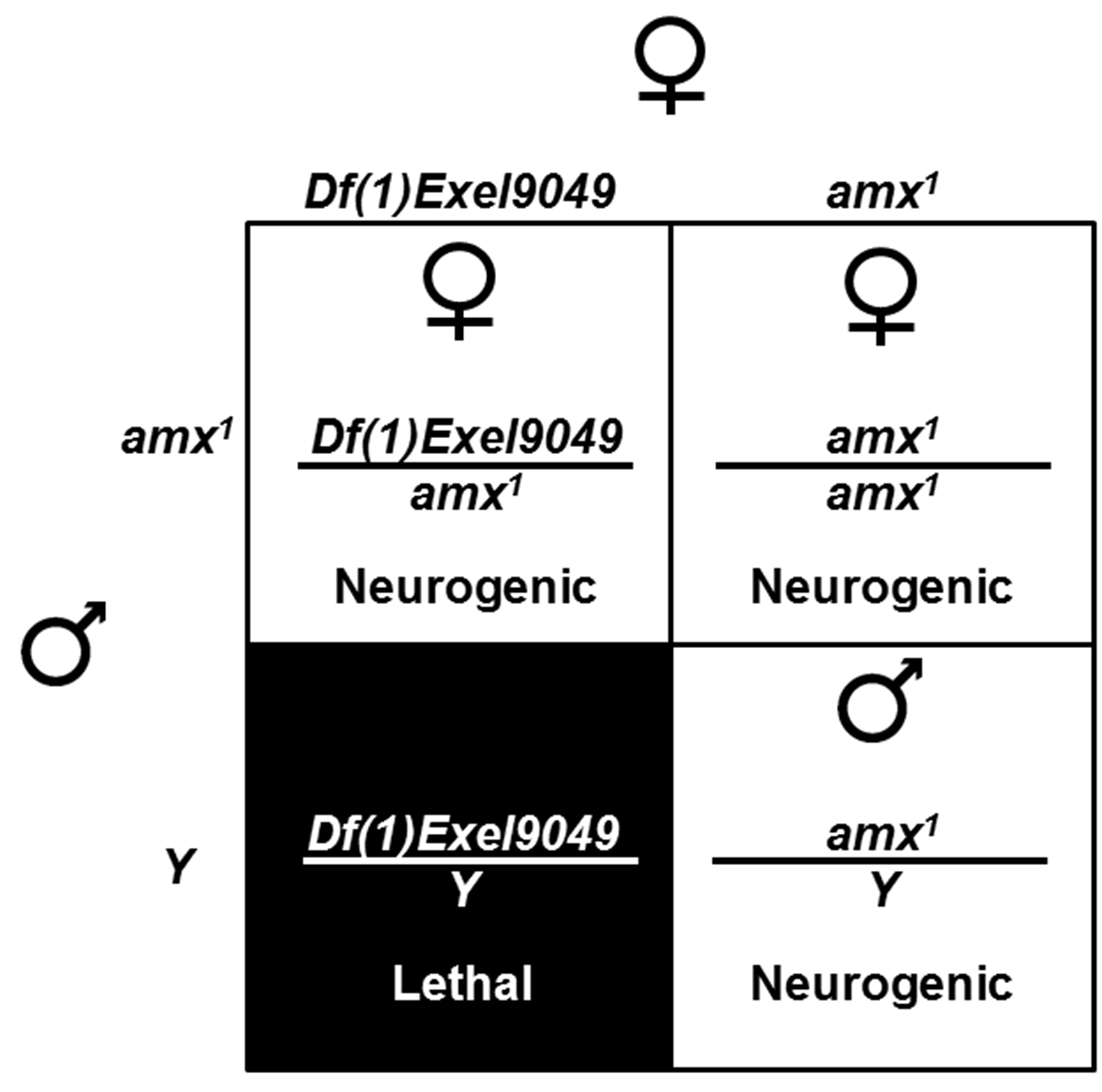

Supplement: S5 Fig — The general crossing scheme is shown for the egg hatching rescue, quantified in Fig 2D. Due to the lethality of Df(1)Exel9049/Y hemizygous male progeny, complete rescue of the amx neurogenic phenotype is expected to lead to a maximum of ~75% egg hatching. (TIFF) [file pgen.1006327.s006.tiff]

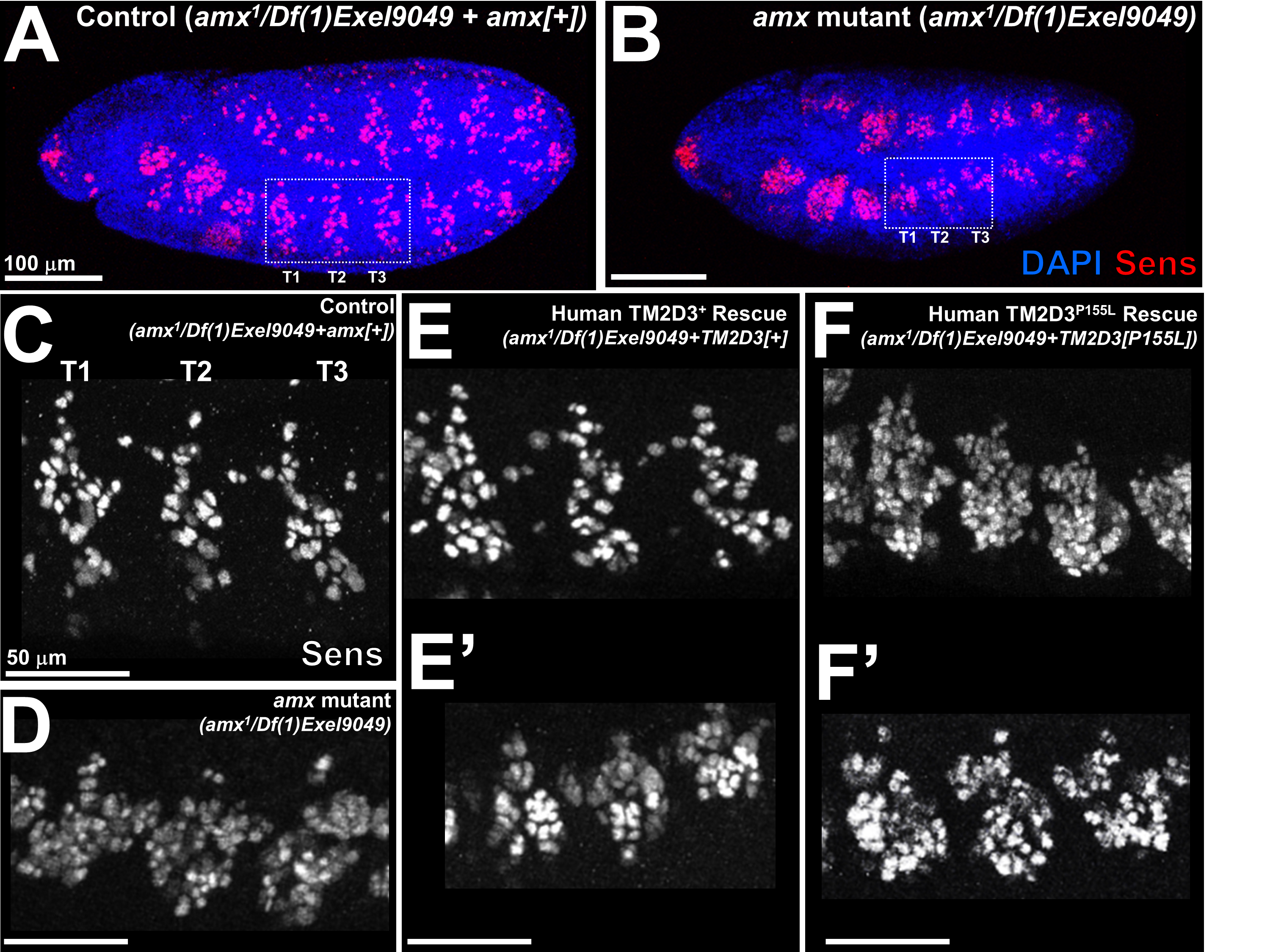

Supplement: S6 Fig — TM2D3[+] but not TM2D3[P155L] can suppress the neurogenic phenotype in the developing fly embryonic peripheral nervous system (PNS). (A-B) Maternal effect neurogenic defects in amx mutants can also be seen in the PNS. Compared to control embryos (A), maternal amx mutant embryos (B) show increased number of Sensory Organ Precursor cells (SOPs), labeled by Senseless (Sens, red), due to defective lateral inhibition. Embryos are counterstained with DAPI (blue). (C-F) PNS phenotypes in embryos laid by amx mutant females with or without amx[+], TM2D3[+], and TM2D3[P155L] genomic rescue constructs. Thoracic segments (T1, T2, T3) of stage 11 embryos are shown. TM2D3[+] can rescue the lateral inhibition defects in SOPs in some (Compare C, D and E) but not all embryos (E’). In contrast, all embryos from amx mutant females with TM2D3[P155L] exhibit neurogenic PNS phenotypes (F, F’). Scale bars = 100μm for A-B, = 50μm for C-F. (TIFF) [file pgen.1006327.s007.tiff]
