# Supplementary material for: Rare Functional Variant in TM2D3 is Associated with Late-Onset Alzheimer's Disease
Source: PLoS Genet. 2016 Oct 20;12(10):e1006327. doi: 10.1371/journal.pgen.1006327 (PMC5072721; doi:10.1371/journal.pgen.1006327)
Supplement: S2 Fig — A schematic of TM2D3 transcripts. Ensembl identifiers (amino acid change due to rs13970957 in parenthesis) are marked by the schematic of each protein-coding transcripts. Bold text highlights transcripts that are also in RefSeq. The exon that contains rs13970957 is marked with an arrow. The schematic was retrieved 27 May 2016 from the Ensembl browser. (PDF) [file pgen.1006327.s003.pdf]

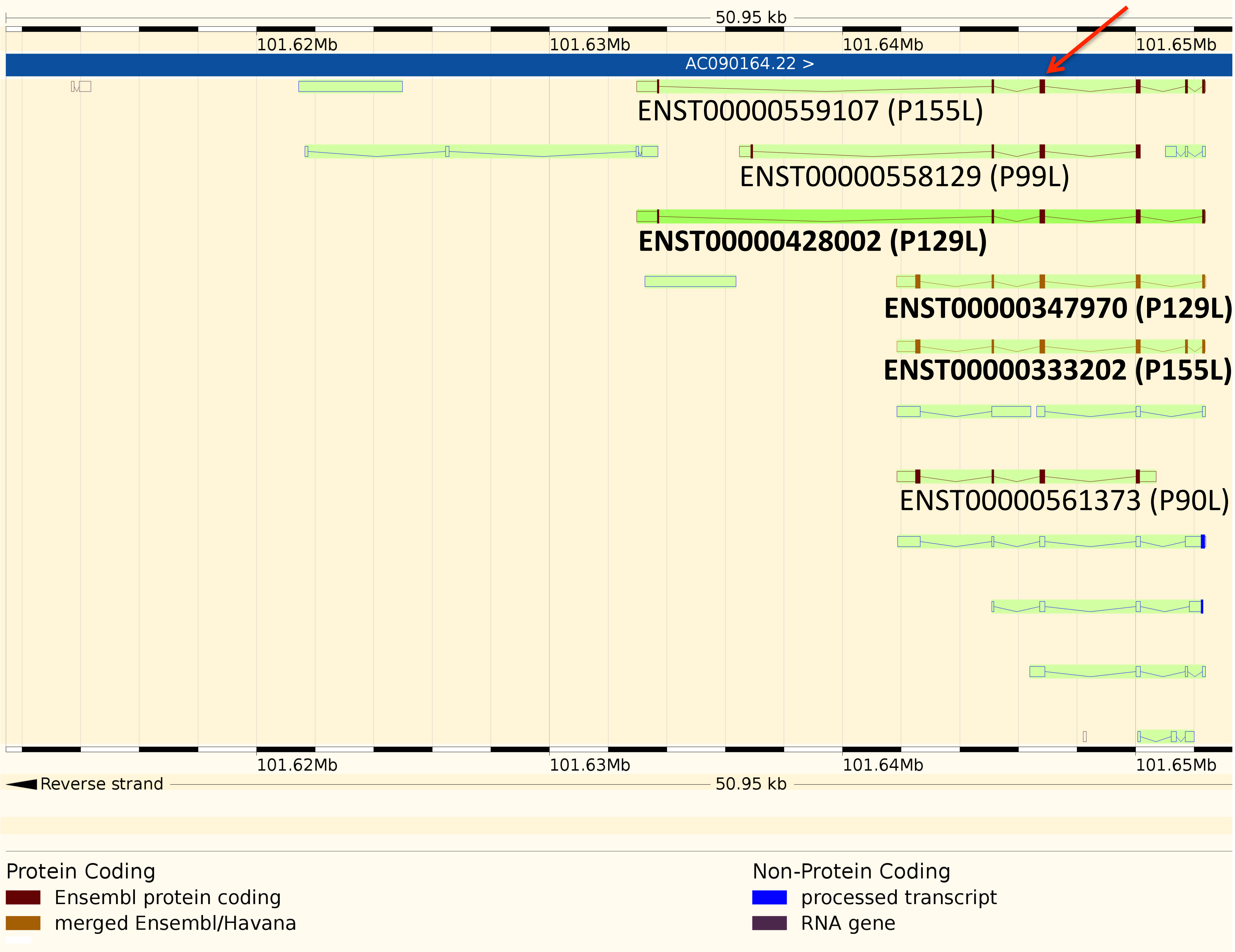

50.95 kb
101.62Mb
101.63Mb
101.64Mb
101.65Mb
AC090164.22 >
ENST00000559107 (P155L)
ENST00000558129 (P99L)
ENST00000428002 (P129L)
ENST00000347970 (P129L)
ENST00000333202 (P155L)
ENST00000561373 (P90L)
101.62Mb
101.63Mb
101.64Mb
101.65Mb
Reverse strand
50.95 kb
Protein Coding
Ensembl protein coding
merged Ensembl/Havana
Non-Protein Coding
processed transcript
RNA gene
