# Supplementary material for: Rare Functional Variant in TM2D3 is Associated with Late-Onset Alzheimer's Disease
Source: PLoS Genet. 2016 Oct 20;12(10):e1006327. doi: 10.1371/journal.pgen.1006327 (PMC5072721; doi:10.1371/journal.pgen.1006327)
Supplement: S3 Fig — Overall gene expression in various tissues, brain tissues are in yellow. TPM (“transcripts per million”) estimated using RSEM [58] on the GTEx data [37] (retrieved 26 May 2016 from UCSC Xena browser). (PDF) [file pgen.1006327.s004.pdf]

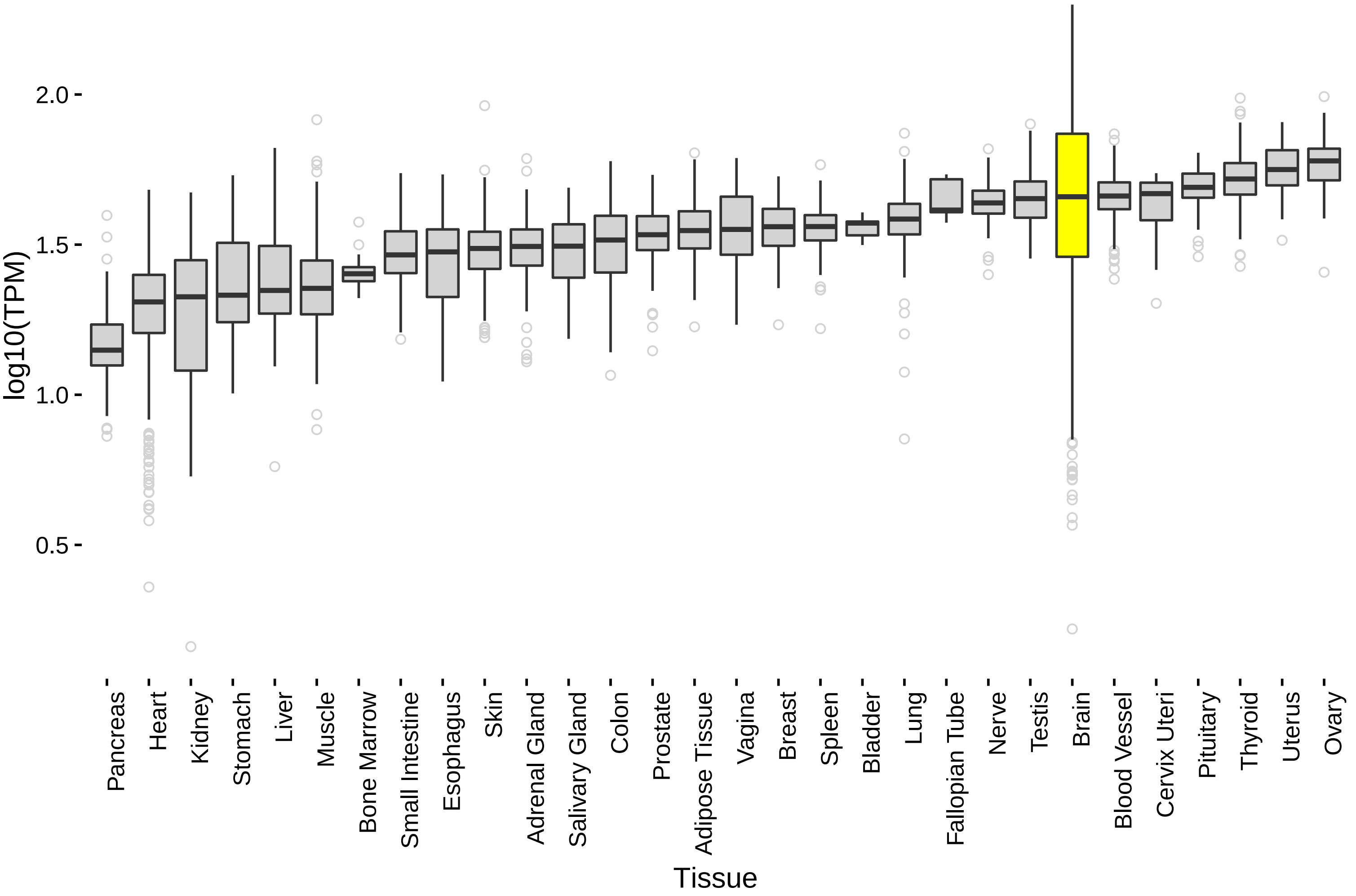
log10(TPM)
2.0
1.5
1.0
0.5
Pancreas
Heart
Kidney
Stomach
Liver
Muscle
Bone Marrow
Small Intestine
Esophagus
Skin
Adrenal Gland
Salivary Gland
Colon
Prostate
Adipose Tissue
Vagina
Breast
Spleen
Bladder
Lung
Fallopian Tube
Nerve
Testis
Brain
Blood Vessel
Cervix Uteri
Pituitary
Thyroid
Uterus
Ovary
Tissue

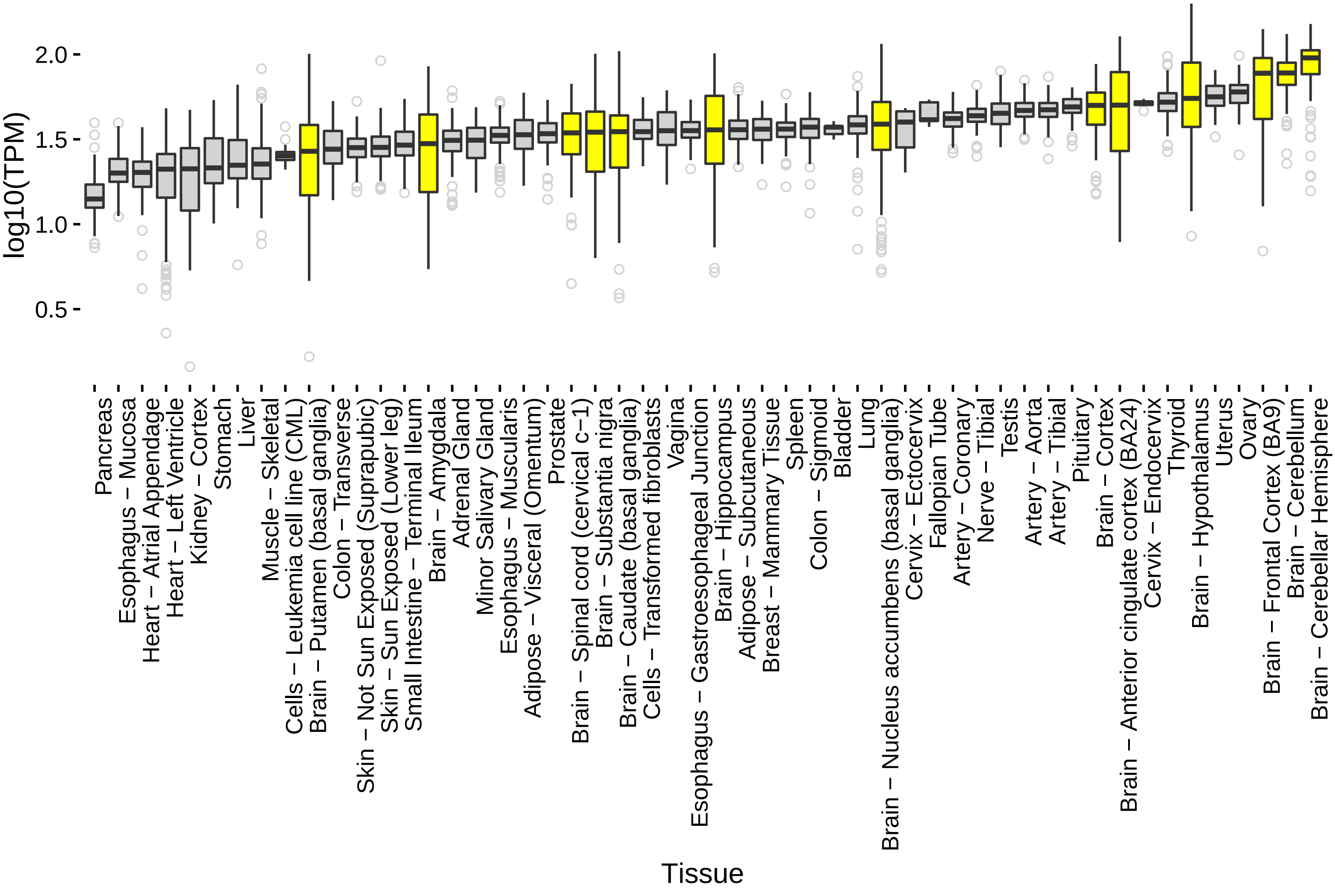
log10(TPM)
2.0
1.5
1.0
0.5
Pancreas
Esophagus – Mucosa
Heart – Atrial Appendage
Heart – Left Ventricle
Kidney – Cortex
Stomach
Liver
Muscle – Skeletal
Cells – Leukemia cell line (CML)
Brain – Putamen (basal ganglia)
Colon – Transverse
Skin – Not Sun Exposed (Suprapubic)
Skin – Sun Exposed (Lower leg)
Small Intestine – Terminal Ileum
Brain – Amygdala
Adrenal Gland
Minor Salivary Gland
Esophagus – Muscularis
Adipose – Visceral (Omentum)
Prostate
Brain – Spinal cord (cervical c-1)
Brain – Substantia nigra
Brain – Caudate (basal ganglia)
Cells – Transformed fibroblasts
Vagina
Esophagus – Gastroesophageal Junction
Brain – Hippocampus
Adipose – Subcutaneous
Breast – Mammary Tissue
Spleen
Colon – Sigmoid
Bladder
Lung
Brain – Nucleus accumbens (basal ganglia)
Cervix – Ectocervix
Fallopian Tube
Artery – Coronary
Nerve – Tibial
Testis
Artery – Aorta
Artery – Tibial
Pituitary
Brain – Cortex
Brain – Anterior cingulate cortex (BA24)
Cervix – Endocervix
Thyroid
Brain – Hypothalamus
Uterus
Ovary
Brain – Frontal Cortex (BA9)
Brain – Cerebellum
Brain – Cerebellar Hemisphere
Tissue
