# Supplementary material for: Rare Functional Variant in TM2D3 is Associated with Late-Onset Alzheimer's Disease
Source: PLoS Genet. 2016 Oct 20;12(10):e1006327. doi: 10.1371/journal.pgen.1006327 (PMC5072721; doi:10.1371/journal.pgen.1006327)
Supplement: S1 Table — Top results of exome-wide single variant (A) and SKAT (B) discovery analysis. Complete results areavailable in dbGAP (accession phs000930). A: Direction = AGES-CHS-FHS-RS, Alleles = non-coding/coding, AF displayed as percentage (%). B: cumAF = cumulative allele frequency displayed as percentage (%), N_SNPs = number of SNPs in test. (PDF) [file pgen.1006327.s008.pdf]

Table S1 A: Top results of exome-wide single-variant discovery analysis. Results from top 10 SNPs shown.
Full results available in dbGAP (accession phs000930)

						Single-variant Test				AF (%) in		
**Variant**	**Gene**	**Chr**	**Position**	**Function**	**Alleles (uncoded/coded)**	**P-value**	**Beta**	**SE**	**Direction***	**All**	**Controls**	**Cases**
rs769449	APOE	19	45410002	intronic	G/A	5.82e-38	0.922	0.072	++++	11.29	10.38	16.62
rs2075650	TOMM40	19	45395619	intronic	A/G	1.32e-24	0.669	0.065	++++	13.64	12.86	18.24
rs6859	PVRL2	19	45382034	UTR3	G/A	1.51e-09	0.264	0.044	++++	40.78	39.96	45.61
rs17154402	SKAP2	7	26765085	nonsynonymous	C/G	2.12e-07	5.508	1.062	0+++	0.03	0.00	0.22
rs139709573	TM2D3	15	102186966	nonsynonymous	G/A	2.02e-06	4.086	0.860	+0-0	0.15	0.14	0.25
rs147960375	C10orf68	10	33137574	nonsynonymous	A/C	4.27e-06	4.474	0.973	++++	0.05	0.02	0.22
rs184982628	HDAC2	6	114265517	nonsynonymous	T/G	4.75e-06	3.268	0.714	0+++	0.07	0.03	0.32
rs146161342	LAMB4	7	107671360	nonsynonymous	T/C	1.29e-05	3.382	0.775	0+++	0.06	0.02	0.29
rs145098571	LRRN4	20	6031509	nonsynonymous	T/C	1.42e-05	4.133	0.952	0++-	0.05	0.02	0.22
rs200181512	EPHA10	1	38184439	nonsynonymous	C/T	1.52e-05	4.055	0.938	0+++	0.05	0.02	0.22

* Order of studies: AGES, CHS, FHS, RS

Table S1 B: Top results of exome-wide SKAT discovery analysis. Results from 10 genes shown.
Full results available in dbGAP (accession phs000930)

			SKAT	Details on SKAT			
**Gene**	**Chr**	**Position**	**P-value**	**# variants in test**	**cumMAF in all (%)**	**cumMAF in controls (%)**	**cumMAF in cases (%)**
SKAP2	7	26839792	4.53e-07	3	0.06	0.02	0.25
TM2D3	15	102189590	8.29e-06	4	0.24	0.24	0.25
ADCY5	3	123052275	6.63e-05	13	0.82	0.80	0.93
SLC6A9	1	44472932	0.000141	6	0.38	0.37	0.47
THOC6	16	3076762	0.000258	8	0.19	0.12	0.61
GGA1	22	38023290	0.000284	5	1.57	1.40	3.15
FOLR3	11	71850463	0.000297	3	0.36	0.28	0.79
CCDC117	22	29178015	0.000334	2	1.35	1.26	1.87
CLMN	14	95678525	0.000369	18	1.00	0.93	1.40
SRBD1	2	45771861	0.00037	24	1.71	1.80	1.17
