# Supplementary material for: Rare Functional Variant in TM2D3 is Associated with Late-Onset Alzheimer's Disease
Source: PLoS Genet. 2016 Oct 20;12(10):e1006327. doi: 10.1371/journal.pgen.1006327 (PMC5072721; doi:10.1371/journal.pgen.1006327)
Supplement: S2 Table — (PDF) [file pgen.1006327.s009.pdf]

Table S2 Variant- and study-specific break-down of *TM2D3, SKAP2,* and *APOE* results

		SKAT	AF		META			AGES		CHS		FHS		RS	
Gene	SNP	(%)[a]	(%)	Annotation	p	beta	se	beta	se	beta	se	beta	se	Beta	se
TM2D3	rs80221524	0.00	0.00	NS											
TM2D3	rs139709573	91.18	0.15	NS	2.02e-06	4.09	0.86	5.09	0.94			-1.15	2.14		
TM2D3	rs145296809	0.00	0.00	NS											
TM2D3	rs151143463	0.00	0.00	NS											
TM2D3	rs150334018	8.76	0.08	NS	0.159	-1.18	0.84	-1.07	2.43	-1.20	1.37	-1.08	2.31	-1.23	1.37
TM2D3	rs146538174	0.03	0.01	NS	0.733	-1.12	3.28			-1.12	3.28				
TM2D3	rs181135440	0.02	0.01	NS	0.76	-1.09	3.58							-1.09	3.58
TM2D3	rs148089973	0.00	0.00	NS											
TM2D3	rs184326854	0.00	0.00	NS											
SKAP2	rs7804356	0.00	25.15	intronic	0.752	-0.02	0.05	-0.03	0.14	0.09	0.08	-0.07	0.12	-0.10	0.09
SKAP2	rs199915571	0.00	0.00	NS											
SKAP2	rs200813179	0.00	0.00	NS											
SKAP2	rs17154402	99.84	0.03	NS	2.12e-07	5.51	1.06			6.16	1.57	5.53	2.60	4.69	1.74
SKAP2	rs1129771	0.00	8.89	NS	0.305	-0.08	0.08	-0.13	0.21	-0.18	0.13	-0.05	0.17	0.04	0.14
SKAP2	rs147926422	0.00	0.00	NS											
SKAP2	rs145659190	0.08	0.01	NS	0.685	-1.17	2.88			-1.17	2.88				
SKAP2	rs137938529	0.08	0.02	NS	0.862	0.22	1.29			-1.14	3.04			0.52	1.42
APOE	rs769449	0.00	11.29	intronic	5.82e-38	0.92	0.07	0.98	0.20	0.74	0.12	1.23	0.19	0.94	0.11
APOE	rs769452	90.52	0.34	NS	0.801	0.09	0.37	-0.13	0.98	0.39	0.65	0.02	1.13	-0.04	0.57
APOE	rs769455	3.22	0.01	NS	0.722	-1.07	3.00					-1.07	3.00		
APOE	exm1479365	6.26	0.01	NS	0.656	-1.20	2.69			-1.20	2.69				
APOE	rs7412	0.00	7.38	NS	0.000732	-0.34	0.10	-0.17	0.26			-0.27	0.20	-0.42	0.13
APOE	exm1479367	0.00	0.00	NS											

a % contribution to the set-based SKAT statistic.
b NS = non-synonymous
