# Supplementary material for: Rare Functional Variant in TM2D3 is Associated with Late-Onset Alzheimer's Disease
Source: PLoS Genet. 2016 Oct 20;12(10):e1006327. doi: 10.1371/journal.pgen.1006327 (PMC5072721; doi:10.1371/journal.pgen.1006327)
Supplement: S3 Table — (PDF) [file pgen.1006327.s010.pdf]

Table S3 Allele frequency of P155L in populations of European ancestry

Cohort	Population	Sample size	MAF (%)
CHARGE[a]	European/European American	40102	0.03
ESP[b]	European/European American	8595	0.06
ExAC[c]	European (non-Finnish)	66720	0.03
ExAC[c]	Finland	8654	0.00
ADGC	US (European American)	14349	0.02
GERAD	UK and Germany	7234	0.06
Generation Scotland	Scotland, UK	20240	0.04
GLACIER	Sweden	965	0.00
DIABNORD	Sweden	928	0.00
FIA3	Sweden	2657	0.00
Finrisk	Finland	15929	0.00

a All, except AGES, European American cohorts who participated in the CHARGE exome chip genotyping included in MAF estimation regardless of phenotype availability [1].
b Exome Variant Server, NHLBI GO, Exome Sequencing Project (ESP), Seattle, WA (URL: http://evs.gs.washington.edu/EVS) [release ESP6500SI-V2, accessed September 2 2015]
c Exome Aggregation Consortium (ExAC) [2], Cambridge, MA (URL: http://exac.broadinstitute.org) [Version 0.3, accessed February 16 2016)].

Referneces

1. Grove ML, Yu B, Cochran BJ, Haritunians T, Bis JC, Taylor KD, et al. Best practices and joint calling of the HumanExome BeadChip: the CHARGE Consortium. PLoS One. 2013;8: e68095. doi:10.1371/journal.pone.0068095
2. Lek M, Karczewski KJ, Minikel E V., Samocha KE, Banks E, Fennell T, et al. Analysis of protein-coding genetic variation in 60,706 humans. Nature. Nature Research; 2016;536: 285–291. doi:10.1038/nature19057
